# Bridging Requirements, Planning, and Evaluation: A Review of Social Robot Navigation

**DOI:** 10.3390/s24092794

**Published:** 2024-04-27

**Authors:** Jarosław Karwowski, Wojciech Szynkiewicz, Ewa Niewiadomska-Szynkiewicz

**Affiliations:** Institute of Control and Computation Engineering, Warsaw University of Technology, 00-665 Warsaw, Poland; jaroslaw.karwowski.dokt@pw.edu.pl (J.K.); wojciech.szynkiewicz@pw.edu.pl (W.S.)

**Keywords:** social robot navigation, human-aware navigation requirements, mobile robot motion planning, social robot perception, quantitative evaluation, benchmarks, human behavior simulation

## Abstract

Navigation lies at the core of social robotics, enabling robots to navigate and interact seamlessly in human environments. The primary focus of human-aware robot navigation is minimizing discomfort among surrounding humans. Our review explores user studies, examining factors that cause human discomfort, to perform the grounding of social robot navigation requirements and to form a taxonomy of elementary necessities that should be implemented by comprehensive algorithms. This survey also discusses human-aware navigation from an algorithmic perspective, reviewing the perception and motion planning methods integral to social navigation. Additionally, the review investigates different types of studies and tools facilitating the evaluation of social robot navigation approaches, namely datasets, simulators, and benchmarks. Our survey also identifies the main challenges of human-aware navigation, highlighting the essential future work perspectives. This work stands out from other review papers, as it not only investigates the variety of methods for implementing human awareness in robot control systems but also classifies the approaches according to the grounded requirements regarded in their objectives.

## 1. Introduction

The presence of robots in populated environments has become broadly discussed in the literature since deployments of interactive museum tour guide robots—*RHINO* [1] and *MINERVA* [2]—in the late 1990s. These field studies have provided many insights, and since then, robot navigation among humans has become a vast field of study.

The field has a historical tradition of being multidisciplinary, with researchers from robotics, artificial intelligence, engineering, biology, psychology, natural language processing, cognitive sciences, and even philosophy collaborating, resulting in a diverse range of outcomes [3,4]. Other than that, social navigation is closely linked to various research topics, such as human trajectory prediction, agent and crowd simulation, and naturally, to traditional robot navigation [5].

One of the primary objectives of robotics is to facilitate the seamless operation of intelligent mobile robots in environments shared with humans [4].

In our work, a *socially navigating robot* is an autonomous machine designed to act and interact with humans in shared environments, mitigating potential discomfort by mimicking social behaviors and adhering to norms. Robot navigation requirements are derived from user studies illustrating human preferences during an interaction, while the robot’s decision-making autonomy relies on perception and planning capabilities.

The range of social robots’ applications is diverse. In the late 2000s, Satake et al. [6] established a field study in a shopping mall where a robot recommended shops to people. A long-term validation of a robot operating in a crowded cafeteria was conducted by Trautman et al. [7]. Another extended deployment was accomplished by Biswas and Veloso [8], whose CoBots reached 1000 km of autonomous navigation. On the other hand, Shiomi et al. [9] performed a short-term validation study of a robot operation in a shopping mall. Recently, social robots are typically utilized for interaction in the context of home assistance and healthcare [3] or deployed for delivery purposes, e.g., pizza, mail, and packages [5].

Despite the recent advancements, mobile robots are still not prevalent in our homes and offices. Mirsky et al. [4] state that a primary factor contributing to this limitation is that achieving full autonomy remains feasible only in controlled environments and typically relies on hard-coded rules or learning from relatively clean datasets.

Our review can be segmented into two perspectives: *requirements* and *algorithmic*. The *requirements* perspective involves exploring various user studies to identify the rules for social robots to adhere to. Our primary focus lies in examining factors that cause human discomfort, as confirmed in real-world experiments involving human participants. In addition to identifying these factors, we aim to extract methods for mitigating discomfort to obtain implementable guidelines for robot control systems. Subsequently, the *algorithmic* perspective categorizes existing research regarding scientific approaches and maps those methods onto specified requirements taxonomy. In summary, our survey stands out by offering an in-depth investigation of aspects often discussed less extensively, while still following the latest developments in navigation.

The remainder of this section explains the scope of the reviewed topics and describes the materials collection methodology. Section 2 reviews previous surveys regarding social robot navigation, whereas Section 3 presents the state of the art from the *requirements* perspective, discussing the conclusions of user studies. The following sections give an *algorithmic* overview on perception (Section 4), motion planning (Section 5), and evaluation (Section 6). The survey proposals explaining identified research gaps are presented in Section 7, while the paper is summarized in Section 8.

### 1.1. Review Scope

The scope of the social robot navigation field is vast, and a comprehensive literature review in every aspect is practically unfeasible. Although we had to limit the scope of topics for a thorough examination, we understand the importance of concepts that could not be covered in this study.

Our survey concentrates on deriving the social robot navigation requirements from literature studies, and, based on that, discusses requirements-driven human-aware robot motion planning and metrics related to the social acceptance of robots. However, this review does not extensively explore the domains of, i.a., explicit communication or negotiation, and the range of interactions investigated was also limited to align with the scope of primary topics.

Effective decision making in socially aware navigation requires communication between robots and humans, particularly when the robot’s knowledge about the environment is limited. Specifically, explicit communication involves the auditory domain, as well as written instructions, which robots should interpret and respond to. Robots also need to convey their intentions and decisions to humans, utilizing verbal and visual techniques such as speech and gestures employing onboard actuators. The topic of explicit communication has been investigated to varying degrees in other review works [4,10,11]. Since it is related to higher-level problem-solving, we decided not to categorize our literature search according to this characteristic. In contrast, implicit communication is commonplace in human–robot interaction studies and is more relevant to the investigated topics; hence, it is widely discussed in our survey, as well as in [4,11,12].

Negotiation in social robot navigation acts as a form of dynamic information exchange. This may involve collaborative decision-making processes, e.g., requesting permission to pass. While the scope of the negotiations field extends way beyond human–robot interaction, this concept has been briefly discussed in other social robotics surveys [11,13].

On the other hand, what substantially affects the requirements and objectives of perception and human-aware robot motion planning is the type of robot. Variations in ground, aerial, or aquatic robots [11,14] significantly impact possible scenarios, hence also the range of human–robot interactions. The taxonomy of our considerations does not differentiate the robot types; instead, we focus primarily on ground-wheeled robots, although some principles and algorithmic techniques may also apply to aerial robots. While mobile manipulators may also fall into the category of ground-wheeled robots, their specific problems of low-level motion control tasks are not investigated.

The physical (contact-rich) interaction between robots and humans is a crucial topic in collaborative robotics and safety management. However, our navigation-focused review examines other types of interactions, namely, unfocused and focused [13], neither of which involve physical contact.

### 1.2. Materials Collection

The chosen methodology of selecting resources included in the survey does not strictly adhere to the scoping strategy typically applied in systematic reviews. Specifically, at first, we conducted a comprehensive literature analysis, drawing from review papers discussed in Section 2. The literature from previous surveys has been confined according to our primary topics and then further supplemented by some crucial works that did not appear in other review papers and more recent citations.

To select newer materials for inclusion in the survey, we searched across IEEE Xplore, ScienceDirect, SpringerLink, ACM Digital Library, and Google Scholar databases, as well as included relevant preprints from ArXiv. The queries used for the search engines were (‘social’ OR ‘human-aware’) AND ‘navigation’ AND ‘robot’, which allowed the gathering of over 600 works from various sources. However, our methodology involved identifying resources (papers, software modules, and datasets) based on their relevance to socially-aware robot navigation and its evaluation methods. Therefore, instead of including the vast amount of results from the databases, we selected the materials based on their appropriateness to the primary topics of the survey. The bibliography was also extended by validation of the cross-references between user studies which also led us to valuable materials. The described selection strategy ensures a concise yet comprehensive review of advancements in the field.

**Figure 1:**
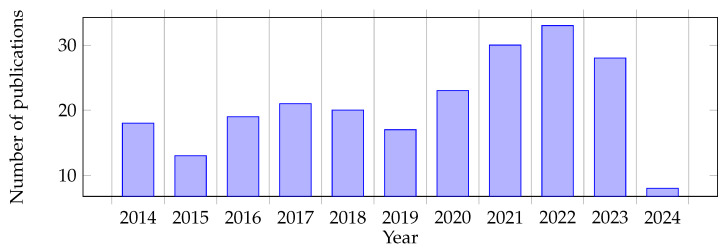
Number of publications from 2014 to 2024 included in the survey by year.

Notably, our survey is also not limited to specific publication years (e.g., [11]) as certain findings, particularly social robot navigation requirements derived from user studies, retain relevance over an extended period. Despite being a subject of research for over 20 years, the field has seen a surge in publications in recent years, as presented in Figure 1.

## 2. Related Work

In recent years, numerous surveys regarding social robot navigation have been proposed [3,4,5,11,12,13,14,15,16,17]. However, the topic is so broad that each one investigates the problem from different perspectives, e.g., evaluation, perception, and hardware.

For example, Kruse et al. [15] discussed the advancements of human-aware navigation for wheeled robots in assistive scenarios. They systematically reviewed the literature, choosing the key features facilitating human-aware navigation as human comfort, robot motions’ naturalness, and sociability. In addition to outlining the basic objectives of social robot navigation, they also focused on spatial constraints that enhance the robot’s sociability. They proposed that integrating them into a single control system mitigates human discomfort. Moreover, they explored numerous methods of human trajectory prediction.

Alternatively, Rios-Martinez et al. [13] delved into sociological concepts regarding the challenges of human-aware navigation. They discussed fundamental concepts related to social conventions and mapped them onto robotics perspectives. In conclusion, they posited that human management of space can be treated as a dynamic system whose complexity extends well beyond proxemics, with contextual factors playing a paramount role in detecting social situations.

In another review paper, Chik et al. [14] offered insights for service robot implementation, highlighting different motion planning system structures for robots operating in populated environments. The discussed navigation frameworks are classified based on their complexity and anticipative potential required for socially acceptable navigation. The authors also provided brief descriptions of algorithms that may enhance social robot navigation and compared them with the traditional methods. Their paper provides practical guidelines on which framework to choose under different conditions.

In a separate study, Charalampous et al. [16] attempted to systematize the recent literature based on the required levels of robot perception for navigating in a socially acceptable manner. They focused on techniques that could allow robots to perceive and interpret their surroundings on a high contextual level. Particularly, they explored methods related to robot’s social awareness (semantic mapping being one of them), the accessibility of datasets, and challenges that need to be confronted when robots operate and interact with humans.

Möller et al. [3] reviewed socially-aware robot navigation, focusing on aspects of computer vision. Namely, their classification of papers is based on the taxonomy of human behavior analysis and modeling, human–robot interaction, active vision, and visual robot navigation. They discussed, i.a., active vision and exploiting it to obtain more data under uncertainty, as well as high-fidelity simulators and numerous datasets, e.g., for human trajectory prediction. The authors pointed out the major research gaps as a lack of formalized evaluation strategies or insufficient datasets and suggested using voice interaction or gesture recognition more commonly to enrich the human–robot interactions.

A more recent survey by Mirsky et al. [4] concentrates on introducing a common language that unifies the vocabulary used in the prior works and highlights the open problems of social navigation. The main topic of the review is conflict avoidance; therefore, the scope of examined papers is bound to works regarding strictly unfocused [13] interactions. As the main challenge of social navigation, they specified standardization of evaluation metrics, group understanding, and context-aware navigation.

Another survey was proposed by Gao and Huang [5], who examined the evaluation techniques, scenarios, datasets, and metrics frequently employed in prior studies on socially aware navigation. They analyzed the drawbacks of current evaluation protocols and proposed opportunities for research enhancing the field of socially-aware robot navigation. Specifically, they stated that there are no standard evaluation protocols to benchmark the research progress, i.e., the field lacks unified datasets, scenarios, methods, and metrics. They also denote the necessity of developing comprehensive instruments to gauge sociability and higher-level social skills during navigational interactions.

Zhu and Zhang [18] discussed *Deep Reinforcement Learning* (*DRL*) and related frameworks for analyzing robot navigation regarding typical application scenarios, i.e., local obstacle avoidance, indoor navigation, multirobot navigation, and social navigation. In turn, Medina Sánchez et al. [19] explored the different aspects of indoor social navigation based on their experience with perception, mapping, human trajectory prediction, and planning. Besides describing the state-of-the-art approaches, they experimented with existing methods and investigated their performance in practice. Guillén-Ruiz et al. [20] discussed recent papers regarding social robot navigation in a more specific context. They reviewed methods for socially aware navigation and classified them according to the techniques implemented in robots to handle interaction or cooperation with humans.

In another recent review, Mavrogiannis et al. [17] synthesized existing problems of social robot navigation and established the core challenges of social robot navigation as motion planning, behavior design, and evaluating the emerging behavior of a robot. Their study aims to diagnose the fundamental limitations of common practices exploited in the field and to provide constructive feedback and suggestions.

Furthermore, at the *Social Navigation Symposium* in 2022, Francis et al. [12] discussed various generic guidelines for conducting social navigation studies and performing valuable evaluation of the experiments. The survey depicts the broadness of the research field and the challenges of social navigation studies. The authors define social robot navigation as respecting the principles of safety, comfort, legibility, politeness, understanding other agents, and being socially competent, proactive, and responsive to context. Their guidelines regard the evaluation of social navigation by the usage of metrics and the development of simulators, scenarios, datasets, and benchmarks. A framework design for this purpose is also presented.

The newest review by Singamaneni et al. [11] examines the field from four perspectives—robot types, planning and decision making, situation awareness and assessment, and evaluation and tools. The survey highlights the broadness of topics and methods involved in social robot navigation. Among their proposals are suggestions for standardizing human actions in benchmarks and establishing unified communication protocols to convey robot intentions.

**Figure 2:**
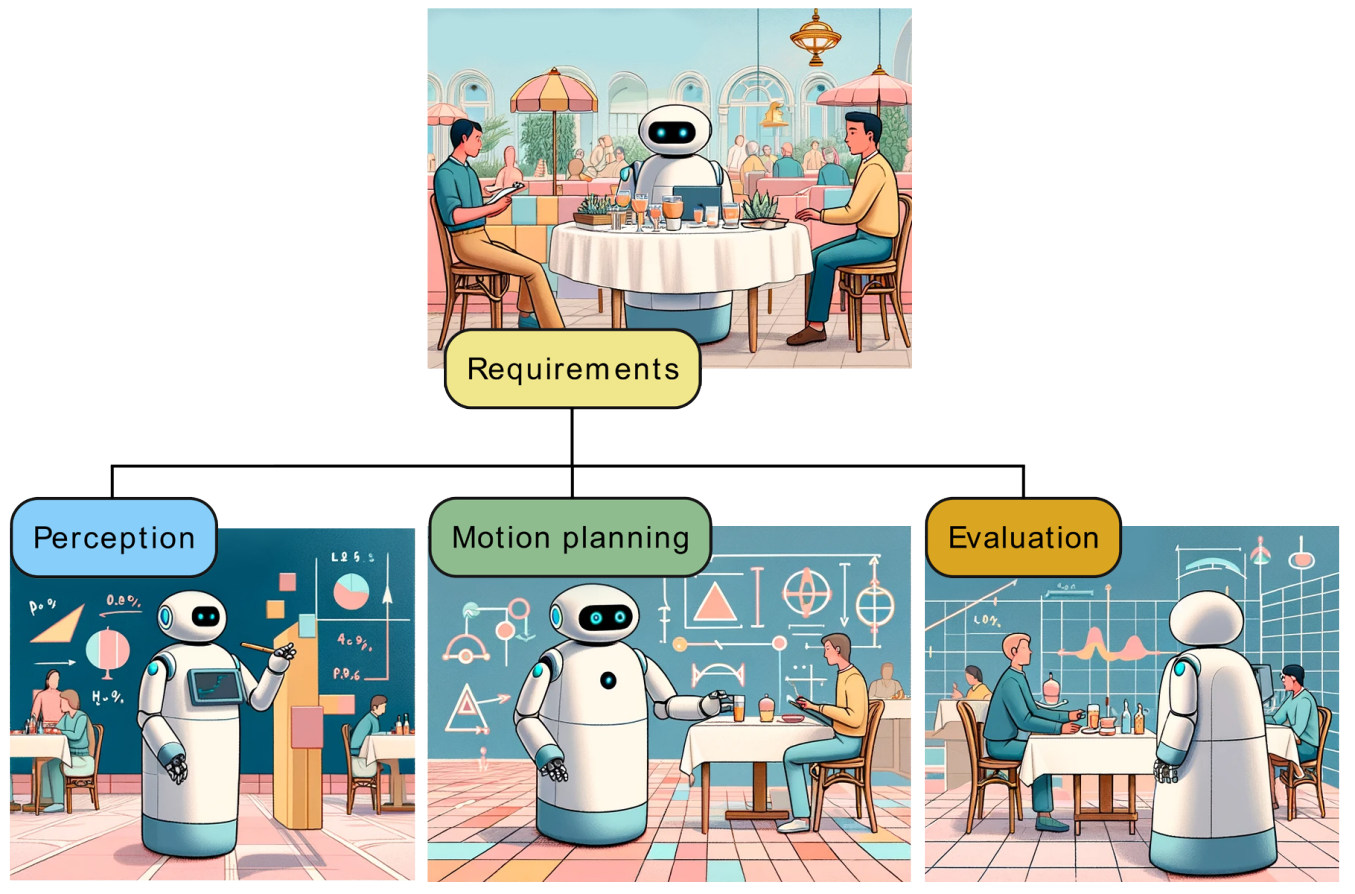
A taxonomy of main concepts in social robot navigation. The principles for perception, motion planning and evaluation are derived from the grounded requirements. Parts of the figure have been generated with the Dall-E AI model.

In contrast to previous review articles, our survey aims to explicitly demonstrate how the key concepts explored by researchers in robotics and social sciences can be transferred into requirements for robot control systems [21] implementing robot navigation tasks. Our review reaches user studies to gather insights and perform the grounding of social robot navigation requirements. After identifying those core principles, perception and motion planning methods are reviewed regarding the taxonomy of requirements Figure 2. The classification of the social robot navigation requirements established in this study enables the identification of the gaps in motion planning algorithms, the drawbacks of state-of-the-art evaluation methods, and the proposal of relevant future work perspectives for researchers in the field. As researchers often try to implement different robot control strategies in an ad hoc manner to mimic human behaviors, we believe that a proper grounding of fundamental features will lead to further developments in the correct direction.

The summary of the state-of-the-art surveys is presented in Table 1, where the varying foci on concepts from perception, through motion planning, to evaluation are visible among different review papers.

## 3. Requirements of Socially Aware Navigation

Social robots were introduced to make human–robot interaction more natural and intuitive [22]. Generic characteristics of social navigation are commonly recalled in review works. For example, Kruse et al. [15] classify the main features as safety, comfort, naturalness, and sociability. On the other hand, in [13], the authors indicate key factors as distinguishing obstacles from persons, considering the comfort of humans—their preferences and their needs, not being afraid of people, and the legibility of motion intentions. More recently, Mavrogiannis et al. [17] proposed a classification that relies on proxemics, intentions, formations, and social spaces, ordered according to the social signal richness. Furthermore, Francis et al. [12] stated that principles of social robot navigation include safety, comfort, legibility, politeness, social competency, agent understanding, proactivity, and contextual appropriateness.

While the aspects above schematically display the goals of social navigation, the authors of the surveys do not attempt to extract the straightforward requirements to follow in social robot navigation. Instead, these terms are loosely defined; hence, they might refer to different means in different contexts or applications. As a consequence, it is tough to determine how to effectively gauge whether the robot behaves in a socially compliant manner. Our survey aims to reduce these abstract terms describing social norms. This is contrary to other review works, where, although taxonomies are presented and articles are classified into those groups, the fundamental concepts persist as vague definitions.

Thus, we perform the grounding of the requirements of social robot navigation. The requirements must be known to properly design a socially-aware robot navigation system. Various techniques have been experimented with an assertive robot, revealing that using knowledge from psychology leads to increased user trust [23]. Incorporating a study-driven approach, we researched human–robot interaction user studies to determine how humans perceive robots navigating around them and how robots should behave around humans under certain controlled conditions. Such an approach allows for obtaining guidelines on how the robot should behave in the presence of humans; hence, precise system requirements can be defined for phenomena that were sufficiently investigated in the literature, while other challenges are coarsely defined.

We separated the study-based grounding of social robot navigation requirements from algorithmic approaches to resolving them. Requirements are obtained from the results of user studies, whereas an algorithmic perspective is presented based on technical papers from the robotics field. Precise requirements grant implementation guidelines and straightforward evaluation of whether the robot behaves as expected.

### 3.1. Taxonomy of Requirements for Social Robot Navigation

Classical robot navigation emphasizes generating collision-free motions for a robot to move to the goal pose as fast as possible. This requires environment sensing for obstacle detection, efficient global pose estimation, and usually map building. Social robot navigation addresses not only the necessities of classical navigation but also extends its capabilities to accommodate social interaction.

**Figure 3:**
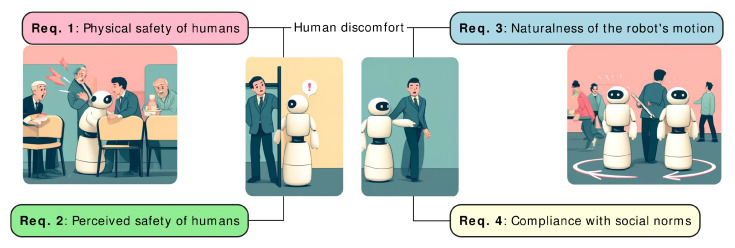
General taxonomy of social robot navigation requirements. The pictures illustrate example concepts of each taxon. The physical safety of humans is related to collision avoidance, whereas the requirements for the perceived safety of humans involve, e.g., avoiding occlusion zones such as corridor corners. Enhancing the naturalness of the robot’s motion links with the avoidance of in-place rotations. Furthermore, compliance with social norms may be connected with certain accompanying strategies. Parts of the figure have been generated with the Dall-E AI model.

The main objective of social navigation is to reduce the human discomfort of the navigating robot. Our taxonomy of social robot navigation requirements (Figure 3) involves the physical safety of humans (**Req. 1**), the perceived safety of humans (**Req. 2**), the naturalness of robot motion (**Req. 3**), and robots’ compliance with social norms (**Req. 4**). Specifically, the perceived safety of humans mostly relies on proxemics theory and the prevention of scaring a human. In turn, the naturalness of the robot’s motion does not affect the safety aspects of humans but regards the trustworthiness of the robot. Lastly, abiding by social conventions focuses on actions and sequences that require rich contextual information to mitigate human discomfort.

Our general taxonomy is designed to classify the essential concepts of social robot navigation clearly and unambiguously into one of the investigated groups to create a generic framework. We expect that the main characteristics selected for the taxonomy will stay pertinent in the future, with the possibility of incorporating additional attributes.

In the remaining part of this section, the social robot navigation requirements are discussed, while the algorithmic concepts describing how these socially aware navigation responsibilities can be embedded into robot control systems are discussed in Section 4 and Section 5.

### 3.2. Physical Safety of Humans (**Req. 1**)

The physical safety of humans is closely related to the collision avoidance capabilities of robots. Social robot navigation inherits this skill from the classical robot navigation requirements.

Francis et al. [12] denote physical safety as the first principle of social navigation that intends to protect humans, other robots, and their environments. The physical safety of humans during navigation is discussed in the newer literature [10,24] but has already been addressed as a fundamental robotics challenge several decades ago [25].

Nonetheless, the physical safety of other robots or machines is also of great significance [17,26,27,28].

For example, Guzzi et al. [29] conducted a study with multiple small-scale robots relying only on local sensing and employing proactive planning integrated with the heuristic pedestrian motion model [30]. In real-world experiments, in a crossing scenario, they observed different frequencies of collisions depending on the sensors’ field of view and safety margin; hence, the collision count was used as one of the metrics for assessing the safety margin parameter. Evaluating *time-to-collision* (*TTC*) is a proactive method to anticipate incoming collisions [31,32] that was also embedded in some benchmarks [33].

### 3.3. Perceived Safety of Humans (**Req. 2**)

**Figure 4:**
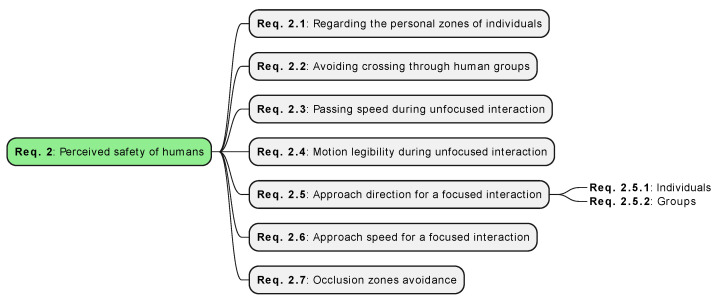
Taxonomy of social robot navigation requirements related to the perceived safety of humans.

The comfort of humans around robots is crucial; however, the robot’s behavior can influence that, potentially causing annoyance or stress [12,15]. Human discomfort during robot navigation often corresponds to a diminished perceived (or psychological) safety of humans. Perceived safety is the factor that might lead to physical safety violations (Section 3.2) if not addressed adequately beforehand. Stress-free and comfortable human–robot interaction is a broad topic [10] influenced by numerous features (Figure 4), including adherence to spatial distancing [13,34], performing natural movements [5], or preventing scaring or surprising a human [15]. The remaining part of this section discusses them in detail.

#### 3.3.1. Regarding the Personal Zones of Individuals (**Req. 2.1**)

Proxemics is the most prominent concept regarding social distancing rules [34,35,36]. Some fundamental studies connected to proxemics theory confirm that the psychological comfort of humans is affected by interpersonal distancing [35,37,38]. Butler and Agah [39] explored the influential factors of how humans perceive a service robot during unfocused interactions. One of them was the distance factor, which induced feelings of discomfort or stress in some configurations. A similar study was conducted by Althaus et al. [40], who validated a navigation system that respects the personal spaces of humans in a real-world study.

Shapes of a personal zone impact the comfortable passing distances. Hall originally specified four circular spaces [34], while the personal zone, reserved for friends, is usually regarded as a no-go zone during unfocused human–robot interaction. Entering the personal zone is counted as a violation of comfort and safety [9,13,41]. The classification of all proxemic zones was described in detail in prior surveys, e.g., [13].

The initially suggested circular shape of the personal space [34] might not appropriately capture the features of human perception and motion. Further empirical studies suggested extending that to an egg shape [42], ellipses [43,44], asymmetrical shapes [45] (prolonged on the nondominant side), or changing dynamically [46]. In [45], it is also reported that the size of personal space does not change while circumventing a static obstacle regardless of walking speed and that the personal space is asymmetrical. The natural asymmetry of personal spaces is also reported in [47], where authors found out that if the robot has to approach a human closely, it is preferred to not move behind a human, so they can see the robot.

Numerous works conducted human-involving experiments to gather empirical data and to model complex and realistic uses of space [48,49,50,51,52]. Participants of the study in [48] rated distances between 1.2–2.4 m as the most comfortable for interaction situations. Experiments by Huettenrauch et al. [53] confirmed that in different spatial configurations, 73–85% of participants found Hall’s personal distance range (0.46–1.22 m) comfortable. Torta et al. [54], in their study involving human–robot interaction, examined the length of comfort zones as specific values of 1.82 m for a sitting person and 1.73 m for a standing person.

Pacchierotti et al. [49,50] examined discomfort as a function of, e.g., lateral distance gap in a hallway scenario. The lateral gap was also examined by Yoda and Shiota [55] in terms of the safety of passing a human by a robot in a hallway scenario. Three types of encounters were anticipated as test cases for their control algorithm, including a standing, a walking, and a running person. They approximated human passing characteristics from real experiments, defining clear formulas to follow in a robot control system. The authors found that the average distance between the passing humans depends on their relative speed and varies from 0.57 to 0.76 m.

The authors of [51] found that the discomfort rates differ between intrusions and extrusions from personal spaces, and distances of approximately 0.85–1.0 m are the most comfortable for a focused interaction with a stranger. On the other hand, Neggers et al. [52] conducted a study similar to [50] and compared their results. They obtained similar outcome and reported that the same function, an inverted Gaussian linking distance and comfort, can be used to fit the results’ data with only a small comfort amplitude shift between [50] and [52]. The authors of [52] also attempted to model an intrusion into personal space as a distance-dependent surface function.

However, there are also diverse exceptions to the mean shape of personal space. For example, Takayama et al. [56] indicated that during the study, participants with prior experience with pets or robots required less personal space near robots compared with people who do not possess such experience. Furthermore, a study presented in [57] endorses the concept that personal space is dynamic and depends on the situation. Velocity-dependent personal space shapes were also considered appropriate in [58,59,60].

Since various studies, even though conducted differently, yield similar results, they seem to approximate human impressions while interacting with robots and, as a consequence, allow modeling of the real-world phenomena of social distancing. The conclusions from the mentioned user studies give insights regarding the implementation of personal space phenomena in robot control systems.

#### 3.3.2. Avoiding Crossing through Human Groups (**Req. 2.2**)

Recent research revealed that pedestrians tend to travel in groups [61,62]. Human groups create focused formations (F-formations) [63]—spatial arrangements that are intended to regulate social participation and the protection of the interaction against external circumstances [13]. F-formations might be static—consisting of people standing together engaged in a shared activity—or dynamic—consisting of people walking together—and might have different shapes [13,63].

The necessity of avoiding crossing F-formations arises from the fact that they always contain an O-space which is the innermost space shared by group members and reserved for in-group interactions. The discomfort caused by a robot to a group might be assessed as the robot’s intrusion into the O-space of the F-formation [64,65]. Results of numerous studies confirm that humans involved in an F-formation keep more space around a group than the mere addition of single personal spaces [66,67,68]; thus, individuals stay away from social groups. Furthermore, research by Rehm et al. [69] found that participants from high-contact cultures stand closer to a group of people compared with people from low-contact cultures.

A general guideline for robots navigating through populated environments is to avoid cutting through social groups [70], but if it is not possible, e.g., in a narrow corridor, they should politely pass through the O-space [12,71].

#### 3.3.3. Passing Speed during Unfocused Interaction (**Req. 2.3**)

Rios-Martinez et al. [13] define unfocused interactions as ‘interpersonal communications resulting solely by virtue of an individual being in another’s presence’. As already highlighted in Section 3.3.1, excessive or insufficient passing speed proved significant in terms of discomfort among humans involved in an unfocused interaction with a robot in numerous experimental studies [39,49,50,60].

The most comprehensive study in that matter was recently proposed by Neggers et al. [60], who assessed human discomfort with a robot passing or overtaking them at different speeds at different distances. They found that higher speeds are generally less comfortable for humans when a robot moves at smaller distances. The authors claimed the inverted Gaussians with variable parameters accurately approximate the experimental results for all combinations of scenarios and speeds. The approximation of their findings with a continuous multivariable function has already been implemented (https://github.com/rayvburn/social_nav_utils (accessed on 20 March 2024)) and can be used for evaluating robot passing speed.

#### 3.3.4. Motion Legibility during Unfocused Interaction (**Req. 2.4**)

Studies conducted by Pacchierotti et al. [50] examined a mutually dynamic situation of passing each other. They assessed human discomfort as a function of the lateral distance gap in a hallway scenario. What they found is that there was no significant impact from the lateral gap size when a robot signaled its passing intentions early. This notion is often referred to as motion legibility, which is an intent-expressive way of performing actions [72]. It can be increased by explicit signaling and also enriching behavior, so it can be used as a cue to the robot intention [73,74].

Lichtenthäler et al. [75] found a significant correlation between the perceived safety and legibility in their study. Gao and Huang [5] considered a flagship example of motion legibility as a scenario where a robot quickly moves toward a person, adjusting its trajectory just before an imminent collision. Despite avoiding direct physical contact, such behavior is likely to produce notable discomfort by the robot heading direction [76] due to lack of early signaling.

#### 3.3.5. Approach Direction for a Focused Interaction (**Req. 2.5**)

Approaching direction to initiate a focused interaction is a broad field of social robot navigation studies. Rios-Martinez et al. [13] describe focused interaction as ‘occurring when individuals agree to sustain a single focus of cognitive and visual attention’. In most experimental cases, focused interaction involves approaching to start a verbal communication or to hand over the transported goods. The taxonomy in this matter separates approaching guidelines between individuals and F-formations.

##### Individual Humans (**Req. 2.5.1**)

In studies conducted by Dautenhahn et al. [77] and Koay et al. [78], participants were seated and asked to gauge their discomfort levels during the handover of objects by a robot that approached from various directions. The subjects of the study preferred frontal approaches over diagonal approaches from the left or right. The contradictory results were found in a study by Butler and Agah [39], where standing participants preferred an indirect approach direction.

Multiple studies depict that human preference is to be approached from the front and within the human field of view [75,79,80,81,82,83,84,85]. Walters et al. [79] examined a robot’s behavior of approaching a human for a fetch-and-carry task. The authors reported that seating participants found the direct frontal approach uncomfortable. The general preference was to be approached from either side, with a preference biased slightly to a rightward approach by the robot. However, the study depicted that a frontal approach is considered acceptable for standing humans in an open area. Another conclusion derived from the study is that humans prefer to be approached from within their field of view; hence approaching from behind should be avoided.

Torta et al. [81] conducted a user study considering different robot approach directions with the final pose at the boundary of a personal space. Similarly, they found that experiment subjects (seated) assessed frontal approach directions (up to ±35°) as comfortable, while they perceived farthermost (±70°) as uncomfortable. Comparable outcomes ensued from the study in [80]. Unlike the results of the user study performed by Dautenhahn et al. [77], in [81], no significant difference was found when the robot approached from the right side or the left side.

Furthermore, Koay et al. [82] researched robot approach distances and directions to a seated user for a handover task. The results show that the preferred approach direction is from either side at a distance of about 0.5 m from the subjects. An interesting fact is that this distance lies within an intimate space [34], but it was preferred because it prevented humans from having to reach out farther with their arms or standing up to pick up the goods from the robot’s tray.

##### Human Groups (**Req. 2.5.2**)

Approaching groups of humans requires slightly different strategies. Ball et al. [84] investigated the comfort levels of seated pairs of people engaged in a shared task when approached by a robot from eight directions. Participants rated robot approach behavior for three spatial configurations of seats. Approaches from common (to all subjects involved) ‘front’ directions were found to be more comfortable (group’s average) than from a shared rear direction. When seated pairs were in a spatial configuration that did not exhibit the common ‘front’ or ‘rear’ direction, no significant statistical differences were found. However, another finding of the study is that the presence and location of another person influence the comfort levels of individuals within the group.

Joosse et al. [85] explored the optimal approach of an engagement-seeking robot towards groups from three distinct countries, employing Hall’s proxemics model [34]. Their findings indicate that the most suitable approach distance seems to be approximately 0.8–1.0 m from the center of the group.

Karreman et al. [83] investigated techniques for a robot to approach pairs of individuals. Their findings revealed a preference among people for frontal approaches (regardless of side), with a dislike for being approached from behind. They also noted that environmental factors appeared to influence the robot’s approach behavior.

#### 3.3.6. Approach Speed for a Focused Interaction (**Req. 2.6**)

Robot speeds are one of the factors impacting discomfort when approaching a human. Since the literature regarding approaching behavior is rich, there are also guidelines to follow in social robot navigation.

Butler and Agah [39] assessed the navigation of a mobile base around a stationary human using various trajectories and equipment resembling the human body. They discovered that speeds ranging from approximately 0.25 to 0.4 m/s were most comfortable, while speeds exceeding 1 m/s were uncomfortable. They also claimed that there might be a speed between 0.4 and 1.0 m/s that produces the least discomfort.

Sardar et al. [86] conducted a user study in which a robot approached a standing individual engaged in another activity. Experiments revealed notable distinctions in acceptance of invading the participant’s personal space by a robot and a human. In their study, only two speeds were evaluated, namely 0.4 and 1.0 m/s, while the robot’s faster speeds were more trustworthy (opposite to human confederates).

In a more recent study, Rossi et al. [87] evaluated speeds of 0.2, 0.6, and 1.0 m/s that affected the robot’s stopping distance while approaching. They found different human preferences for stopping distance depending on the activity currently executed by humans. Sitting participants favored shorter distances while walking subjects longer ones.

#### 3.3.7. Occlusion Zones Avoidance (**Req. 2.7**)

Occlusion zones are related to areas not reached by the robot’s sensory equipment. Despite the robot’s most recent assumptions suggesting that these areas were previously unoccupied, such estimates may be inaccurate. Consequently, robots should avoid traversing near blind corners, as they may fail to detect individuals behind them, and vice versa. By going around the corner with a wider turn, the robot can explore the occluded space earlier, making it possible to react to humans sooner [15]. Proactivity in that matter prevents surprise or panic and generally positively impacts comfort and physical safety.

User studies generally confirm this issue, showing that humans tend to shorten their paths [88,89] to minimize energy expenditure. Taking shortcuts in public spaces increases the risk of encounters around blind corners.

Francis et al. [12] suggested that a robot entering a blind corner should communicate intentions explicitly with voice or flashing lights. However, this seems slightly unnatural, as even humans avoid shouting in corridors. Enabling audio or flashing lights might also be annoying for surrounding workers in shopping aisles.

### 3.4. Naturalness of the Robot Motion (**Req. 3**)

**Figure 5:**
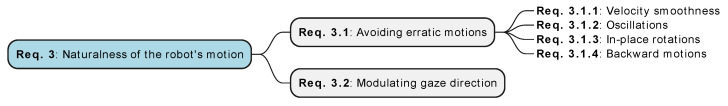
Taxonomy of social robot navigation requirements related to the naturalness of the robot’s motion.

The naturalness of a robot’s motion can be referred to as emerging robot behaviors that are not perceived as odd. This is often related to the avoidance of erratic movements and oscillations Figure 5. Keeping a smooth velocity profile also produces an impression of trust and legibility among observing humans [75].

#### 3.4.1. Avoiding Erratic Motions (**Req. 3.1**)

Erratic motions involve sudden changes in velocity, making it difficult to anticipate the next actions. This term is often used to describe the behavior of objects exhibiting chaotic movement patterns that make the robot look confused.

Erratic motions are often related to the smoothness of a robot’s velocity profile (**Req. 3.1.1**). Natural motions favor movements with minimum jerk [90], with mostly stable linear velocity and the angular velocity of zero, i.e., adjusting orientation only when necessary [5,15].

In contrast to smooth velocities, oscillating motions (**Req. 3.1.2**) involve alternating forward and backward motions, where the robot effectively does not make any progress. They may be present in some navigation approaches that rely solely on *Artificial Potential Field* [91] or *Social Force Model* [43].

Additionally, the in-place rotations (**Req. 3.1.3**) of a robot appear unnatural for human viewers; hence, it is preferred to avoid trajectories where a turning is performed on one spot [90,92]. Also, significant backward movements (**Req. 3.1.4**) should be avoided, as individuals rarely move in reverse in public areas. Such actions can pose collision risks, particularly for mobile bases lacking range sensors at the back.

#### 3.4.2. Modulating Gaze Direction (**Req. 3.2**)

A broad area of research regarding motion naturalness corresponds to modulating the robot gaze direction. Humanoid robots are typically equipped with a ‘head’, inside which a camera is located (*RGB* or *RGB-D*), e.g., Nao, TIAGo, Pepper, Care-O-bot. Pan and tilt motions of the head joints can be used to modulate gaze direction.

Gaze direction is considered one of the social signals (cues) and a specific type of nonverbal communication between a robot and surrounding humans [4]. Among humans, it is closely related to their perception captured by the notion of *Information Process Space* [13]. Gaze is a general concept in which measurable aspects can be evaluated, such as fixation count and length [93], as well as gaze–movement angle [94]. Both provide valuable insights into human trajectory or behavior prediction [4].

##### Unfocused Interaction

In a study by Kitazawa and Fujiyama [95], the authors investigated gaze patterns in a collision avoidance scenario with multiple pedestrians moving along a corridor. Results of the experiment show that humans pay significantly more attention to the ground surface, which they explain as a focus on detecting potential dynamic hazards than fixating on surrounding obstacles. In an experiment conducted by Hayashi et al. [96], they noticed that participants were more willing to speak to the robot when it modulated its gaze direction. Kuno et al. [97] also concluded that robot head movement encourages interaction with museum visitors.

Fiore et al. [98] analyzed human interpretations of social cues in hallway navigation. They designed a study to examine different proxemics and gaze cues implemented by rotating the robot sensors. The results depict that the robot’s gaze behavior was not found to be significant, contrary to the robot’s proxemics behavior that affected participant impressions about the robot Section 3.3.1. Similarly, a study by May et al. [99] showed an understanding of robot intentions while conveyed using different cues. It turned out that the robot was understood better when a mechanical signal was used compared with using the gaze direction cue. Also, Lynch et al. [100] conducted a study employing a virtual environment where virtual agents established a mutual gaze with real participants during path-crossing encounters in a virtual hallway. Subjects of the study found the gaze factor to not be important for inferring about the paths of the virtual agents.

Different strategies of gaze modulation were studied by Khambhaita et al. [101]. Their research indicates that the robot’s head behavior of looking at the planned path resulted in more accurate anticipation of the robot’s motion by humans compared with when the head was fixed. The authors also found that the robot operating with the head behavior of alternately looking at the path and glancing at surrounding humans gave the highest social presence measures among the subjects. Similarly, Lu et al. [102] discussed a strategy of a robot looking at the detected human followed by looking ahead in 5-second cycles.

##### Focused Interaction

Research has shown that gaze modulation of the robot’s focused interactions should be treated differently than unfocused ones. Breazeal et al. [103] explored the impressions of humans participating in an experiment with a Kismet robot capable of conveying intentionality through facial expressions and behavior. They identified the necessity of gaze direction control for regulating conversation rate, as the robot directs its gaze to a locus of attention.

In another study, Mutlu et al. [104] implemented a robot gaze behavior based on previous studies [105,106] and their observations that people use gaze cues to establish and maintain their conversational partner’s roles as well as their own. The gaze behavior strategy produced turn-yielding signals only for conversation addressees. In their experiment, they found that using only the gaze cues, the robot manipulated who participated in and attended to a conversation.

### 3.5. Compliance with Social Norms (**Req. 4**)

**Figure 6:**
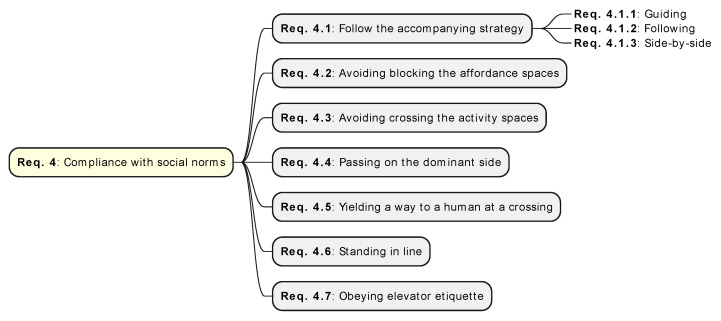
Taxonomy of social robot navigation requirements related to the robot’s compliance with social norms.

Navigating humans adhere to diverse social norms influenced by cultural, interactional, environmental, and individual factors such as gender and age. Therefore, the robot’s compliance with social conventions is also a multifaceted concept (Figure 6), in contrast to low-level motion conventions, such as approach velocity. The aforementioned factors shape high-level social conventions involving navigation-based interactions like queuing, elevator decorum, yielding way to others, and adhering to right-of-way protocols. Robots considered sociable abide by social conventions. Despite the existence of customary routines, they are often challenging to model precisely due to their abstract nature, as seen in the discussion by Barchard et al. [107].

The authors of surveys [5,15] exemplify that even if the robot’s movements may appear natural and unobtrusive (**Req. 3**), it can violate typical social conventions. For instance, entering a crowded elevator without allowing occupants to exit first breaches common expectations, thereby potentially causing discomfort. Also, in different user studies, it is reported that human discomfort can be caused due to violations of social norms even if the rules of perceived safety of humans are properly adhered to in the robot navigation [108,109].

There are no predetermined sets of high-level social conventions, making compliance a dynamic and context-dependent aspect of robotic behavior [5] that requires a diverse level of contextual awareness.

The most common and meaningful social conventions examined in the literature are illustrated below. The complementary discussion attempts to clarify how they should be addressed in robot control systems.

#### 3.5.1. Follow the Accompanying Strategy (**Req. 4.1**)

Strategies of executing the task of accompanying humans by the robot are dictated by the social conventions of how humans navigate in relation to other pedestrians. Customary human behaviors entail how robots should adjust their movements based on the relative position of the accompanying human (or humans), ensuring smooth and natural interactions.

##### Tracking Humans from the Front (**Req. 4.1.1**)

Numerous studies reviewed the relative pose that the robot should maintain while tracking a human from the front. For example, Jung et al. [110] performed a study to evaluate how often humans look back at the robot that tracks the subject from behind. They found that participants often looked back as they were curious about the robot, whether it bumped into them or tracked them well. The authors concluded that tracking from the front might be more comfortable and designed a robot control strategy that involves moving 1 m ahead of the tracked human, whose local movement goal is inferred by the robot online.

On the other hand, Young et al. [111] compared various relative poses for a robot led on a leash by a participant. The results reveal that having the robot move in front of the person was the most comfortable approach for joint motion. In another study, Carton et al. [112] proposed a framework for analyzing human trajectories. Their studies led to the conclusion that humans plan their navigation trajectories similarly whether they are walking past a robot or another human.

##### Person Following (**Req. 4.1.2**)

Gockley et al. [113] evaluated methods of avoiding rear-end collisions of a robot following a person. The first approach focuses on direction-following, where the robot follows the heading of a person, whereas the second method, path-following, relies on imitating the exact path that a person takes. The participants of the real-world experiments rated the direction-following robot’s behavior as substantially more human-like. However, the participants rated that the robot stayed too far away (1.2 ± 0.1 m) from them while moving.

Following an individual in populated environments is challenging as crowd behavior often manifests as flows of social groups, with individuals typically following the flow [61]. Studies show that joining a flow with a similar heading direction is more socially acceptable, resulting in fewer disturbances to surrounding pedestrians [114]. Collision avoidance techniques for following one person through a populated environment are discussed in [115,116].

##### Side by Side (**Req. 4.1.3**)

The tendency for people to walk side by side when walking together was discussed by Kahn et al. [117]. In situations with only two individuals walking, they typically adopt a side-by-side formation, while in crowded conditions or with three or more individuals, more complex formations such as ‘V’ shapes are observed [118]. Spatial preferences of humans when being followed by a robot were reviewed in [119]. In the majority of studies, the robot’s relative position to the person typically remains constant, with any adjustments being made primarily in response to environmental factors.

Saiki et al. [120] discussed how robots can serve walking people. In their experiments, people trajectories were recorded to develop a histogram of relative distances. The conclusion is that people’s average distance while walking alongside each other is 0.75 m.

Karunarathne et al. [121] designed a spatial model for side-by-side accompaniment without explicit communication about the goal of a human. During their study, they found that the distance maintained in a robot–human pair (1.25 m) was larger than that of the human pair on average (0.815 m).

#### 3.5.2. Avoiding Blocking the Affordance Spaces (**Req. 4.2**)

The concept of affordance space relates to the potential activities that the environment offers to agents [122]. Affordance spaces could be mapped as free regions or banned regions in a function of time [123]. They have no specific shape [13] as they depend on specific actions.

Affordance spaces are specific to the robot environment and can be exemplified by the area near a painting in a gallery or menu stands in restaurants. In general, an affordance space can be crossed without causing disturbance to a human (unlike activity spaces in Section 3.5.3), but blocking an affordance space could be socially not accepted [13]. Also, for robots with a limited *field of view* (*FOV*), it is essential to utilize a predefined map of affordance spaces.

Raubal and Moratz [124] discussed a robot architecture incorporating a functional model for affordance-based agents. The crucial concept is to consider the information about locations of affordance spaces when selecting a coarsely defined (region-based) navigation goal or a goal on a topological map. The notion of affordance spaces was also discussed in the context of learning them online [125], as well as in gaining knowledge from the analysis of human trajectories [126].

#### 3.5.3. Avoiding Crossing the Activity Spaces (**Req. 4.3**)

The activity space is an affordance space linked to an ongoing action performed by an agent—a human or another robot [13]. An activity space can be exemplified by the area between an observer and a painting in a gallery. Once the visitor initiates this space, the robot is obliged not to cross it [122]. Additionally, the robot’s perception has to dynamically infer whether a certain agent has initiated an activity space, e.g., by observing an object [125]. Furthermore, the activity space should be conditionally constrained; for instance, it should be less restrictive for a shorter robot compared with a taller one that might fully occlude the painting when crossing through an activity space.

#### 3.5.4. Passing on the Dominant Side (**Req. 4.4**)

Bitgood and Dukes [89] discussed that people tend to proactively move to the right half portion of a hallway or a narrow passage, which is tied to cultural traffic rules. Multiple existing social robot navigation approaches already implemented strategies to follow the right side of the corridor or to favor passing humans on the right [59,73,116,127]. However, as Bitgood and Dukes suggest, this might not be a strict rule to follow in crowded spaces, as some people follow the other side as they have an incoming left-turn destination [89]. This is supported by the study conducted by Neggers et al. [60], who also examined the effect of the passing side and found that participants reported equal comfort levels for both sides. Nevertheless, Moussaïd et al. [128] conducted a set of controlled experiments and observed pedestrians’ preference to perform evasive maneuvers to the right while passing each other.

#### 3.5.5. Yielding the Way to a Human at Crossings (**Req. 4.5**)

Moller et al. [3] posed the problem of who goes first at an impasse as one of the social conventions that are ‘less well-defined’. As stated in a survey by Mirsky et al. [4], the term ‘social navigation’ usually refers to a human-centric perspective; therefore, the robot is often obliged to yield the way to a human at a crossing.

The user study performed by Lichtenthäler et al. [75] showed that in the crossing scenario, the participants favored the navigation method in which the robot stopped to let a person pass. Yielding the way to a human based on the predicted motion was also investigated in [65].

#### 3.5.6. Standing in Line (**Req. 4.6**)

Standing in line while forming a queue is one of the most common collective behaviors of humans. Nakauchi and Simmons [129] modeled how people stand in line by first collecting empirical data on the matter. Further, they utilized these data to model a range of behaviors for a robot tasked to get into a queue, wait, and advance in the queue alongside other individuals awaiting service.

#### 3.5.7. Obeying Elevator Etiquette (**Req. 4.7**)

‘Elevator etiquette’ refers to the customary rules of humans entering and exiting a bounded space through a doorway, specifically letting people leave an elevator before attempting to enter. These rules are generalizable to numerous closed areas like rooms and corridors.

Gallo et al. [130] proposed the machine-like approach for the design of robot behavior policies that effectively accomplish tasks in an indoor elevator-sharing scenario without being disruptive. Alternatively, Lin et al. [109] discussed the social appropriateness of lining up for an elevator in the context of deploying a mobile remote presence. Elevator-related conventions were tackled in a robotic competition—“Take the Elevator Challenge” [131].

### 3.6. Discussion

We acknowledge that the proposed set of primitive requirements is subject to extension as the social navigation studies advance and new issues or additional cases are found [12]. Not only have some requirements mentioned above not been sufficiently studied, but there are also many other human conventions that have not been considered at all in user studies with robots; hence, there are no clear guidelines on how they can be tackled properly in social robot navigation. As a consequence, the comprehensive method for assessing compliance with social norms remains unresolved, in contrast to the agreement on criteria for evaluating the physical and perceived safety, as well as most cases covered by naturalness aspects.

An example phenomenon that was not targeted by user studies to the extent that allows establishing specific principles is facial expressions. Petrak et al. [71] discussed a side note of their study that enhanced robot facial expressions and gestures could make the behavior easier to anticipate for the experiment participants. Kruse et al. [15] pointed out additional navigation conventions, such as giving priority to elderly people at doorways, asking for permission to pass, and excusing oneself when one has to traverse a personal zone to reach a goal. Furthermore, Gao and Huang [5] indicated observing right-of-way at four-way intersections as another navigation-based interaction. On the other hand, despite that overtaking on the nondominant side has been implemented in some navigation methods [59,132], there are no clear guidelines that such behavior is common in environments other than narrow passages.

Nevertheless, implementing all requirements in a single robot control system is an enormous challenge, while integrating all constraints and norms requires rich contextual awareness of the robot.

## 4. Perception

Robot perception plays a substantial role in safe navigation and expands the intelligence of a robot. Social robots must differentiate obstacles from humans to interact in a discomfort-mitigating manner.

In robotics, various types of exteroreceptors [21] are utilized to perceive the environment. Tactile sensors provide feedback about physical contact, enabling robots to detect and respond to touch [40,49,50,133,134]. They are crucial for tasks requiring object recognition that other sensor types cannot capture. Sonar sensors utilize sound waves to detect the presence, distance, and velocity of objects, allowing robots to navigate and avoid obstacles in dynamic environments [39,40,135,136,137]. Laser range finders use laser beams to measure distances accurately, aiding in mapping and localization tasks [49,138,139,140,141,142,143]. *RGB* cameras capture images in visible light, enabling robots to recognize objects, navigate environments, and interpret visual cues [27,40,144]. Finally, *RGB-D* cameras, equipped with depth sensors, provide both color and depth information, enhancing object detection and enabling 3D mapping [140,145,146,147]. These sensor types play essential roles in robotics research and development, enabling robots to perceive and interact with their surroundings effectively.

**Figure 7:**
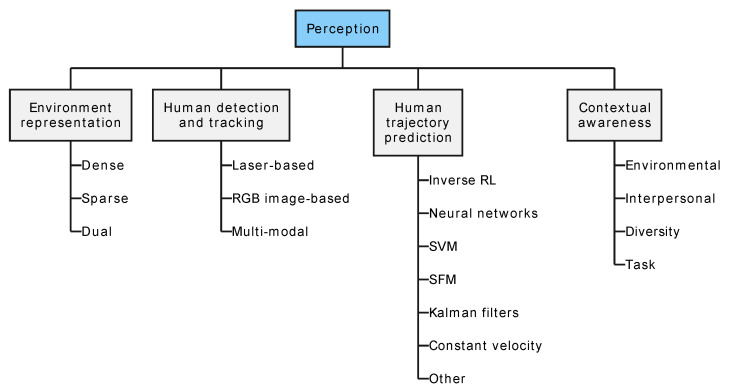
A taxonomy of perception for social robot navigation.

The remainder of this section follows the taxonomy illustrated in Figure 7.

### 4.1. Environment Representation

Besides detecting obstacles and tracking humans, robot perception is usually employed to collect subsequent observations of the surroundings to create an environment model, among which the most popular are dense, sparse, and dual representations.

A dense representation constitutes a discretized map of the robot environment. Classical maps contain all types of obstacles embedded into the environment model without a semantic distinction. The most common planar map types are occupancy grids [148] and costmaps [149], while octomaps [150] represent occupancies in 3D space. The pioneering dense model is an occupancy grid [148] that represents the environment as a binary grid (graph) where each cell is either occupied or free, and all occupied cells are treated as equal obstacles. Therefore, costmaps were proposed to extend the classical occupancy grids. Costmaps introduce intermediate states (between free and occupied) of a cell [149] and constitute a 2D traversability grid in which cells are given a cost of traversal reflecting the difficulty of navigating the respective area of the environment [151]. This allows robots to plan paths that optimize not just for avoiding collisions but also for factors like proxemics. The dense representation of an environment is often solely used in classical robot navigation approaches [138,150,152].

Sparse environment representations typically refer to representations where only certain key features or landmarks are represented explicitly, with the rest of the space left unstructured or minimally represented. Sparse representation usually provides a concise description of the objects detected in the environment, constituting their semantic information with geometric attributes [28,153,154,155]. This method of storing environment objects also allows, e.g., applying linear algebra formulas to easily predict objects’ motion.

Dual environment representations, combining dense and sparse ones, are commonly used in social robot navigation [156,157,158,159]. While obstacle-filled costmaps are calculated, robot perception modules simultaneously detect and track humans in the environment. They provide sparse data about each human, e.g., a pose and velocity, or even spatial relationships [140,160]. Such information allows for dynamic modeling of personal spaces of individuals (**Req. 2.1**) and O-spaces of F-formations (**Req. 2.2**), which can later be embedded onto layered costmaps [161]. Layered costmaps extend the notion of traditional costmaps to facilitate separate representations of different contextual cues as spatial constraints in the robot environment. The resultant costmap with enriched information is flattened for motion planning; therefore, classical algorithms can still be used.

### 4.2. Human Detection and Tracking

Social robot navigation encompasses the awareness of humans surrounding the robot, as they must be treated differently from typical obstacles. The awareness arises from detecting and tracking people by the robot perception system [115] as well as exhibiting behavior that mitigates the discomfort of nearby humans. Various methods for human detection and tracking have been proposed in the literature [140,162,163,164,165,166,167].

Arras et al. [162] proposed a method utilizing a supervised learning technique for creating a classifier for people detection. Specifically, *AdaBoost* was applied to train a classifier from simple features of groups of neighboring beams corresponding to legs in the *LiDAR*’s range data. Similarly, Bozorgi et al. [167] focused on *LiDAR* data filtering to obtain robust human tracking in cluttered and populated environments. They integrated Hall’s proxemics model [34] with the global nearest neighbor to improve the accuracy of the scan-to-track data association of leg detection. Results of their experiments show that their method outperformed the state-of-the-art detector from [163].

In contrast, Linder et al. [140] proposed a multimodal (*LiDAR* and *RGB-D*) people-tracking framework for mobile platforms in crowded environments. Their pipeline comprises different detection methods, multisensor fusion, tracking, and filtering. Triebel et al. [160] extended the multihypothesis tracker from [168] to detect F-formation arrangements. Both works were integrated and implemented in the *SPENCER* robot [140,160].

Redmon et al. [164] framed the object detection problem as a regression problem to spatially separated bounding boxes and associated class probabilities. They proposed a generic framework for detecting objects of various classes on 2D images. Alternatively, Cao et al. [166] proposed an *Open-Pose* system for human skeleton pose estimation from *RGB* images. In another work, Juel et al. [169] presented a multiobject tracking system that can be adapted to work with any detector and utilize streams from multiple cameras. They implemented a procedure of projecting *RGB-D*-based detections to the robot’s base frame that are later transformed to the global frame using a localization algorithm.

Theodoridou et al. [144] used *TinySSD* [165] for human detection in their robot with limited computational resources. *TinySSD* is a lightweight single-shot detection deep convolutional neural network for real-time object detection, which only finds people in the images; hence, the authors of [144] had to perform image and range-based data matching in their system.

In real-world studies, robot sensors are used to detect and track humans. The survey by Möller et al. [3] discusses, i.a., the active perception idea. The authors denoted that active vision systems can influence the input by controlling the camera. As an extension of active perception, they depict active learning [170], which also influences the input data but during the training process. This enables the agent to intelligently choose what data points to exploit next.

To the best of our knowledge, currently, the most comprehensive human perception stack is *SPENCER* [140,160], which is available as the open-source software (https://github.com/spencer-project/spencer_people_tracking (accessed on 20 March 2024)) compatible with the *Robot Operating System* (*ROS*) [171,172].

### 4.3. Human Trajectory Prediction

In social navigation, classical planning methods, e.g., *Artificial Potential Field* (*APF*) [91] or *DWA* [135] often exhibit limited efficacy as pedestrians are treated merely as uncooperative obstacles. This limitation is exemplified by the freezing robot problem [173], where a mobile robot may become immobilized in a narrow corridor when confronted with a crowd of people unless it can anticipate the collective collision avoidance actions [174]. Therefore, predicting human trajectories is one of the fundamental concepts in social robot navigation, in particular in unfocused human–robot interactions, where explicit communication between agents is not present. Understanding how agents move can reduce the potential for conflicts, i.e., sudden encounters in which humans and robots might collide (**Req. 1**) [4,175]. Another particularly important aspect is that humans frequently undergo lengthy occlusion events; hence, their motion prediction prevents possible unexpected encounters.

In the social robot navigation literature, the prevailing method is the *Inverse Reinforcement Learning* (*IRL*) [176], which is based on the *Markov Decision Process* (*MDP*) [177]. The *IRL* identifies reward functions based on the observed behavior, enabling robots to learn from human demonstrations. It can be classified as an offline inference and learning method [4]. Henry et al. [178] used *IRL* to learn human motion patterns in simulation to use them later for socially aware motion planning. Rhinehart et al. [179] extended *IRL* for the task of continuously learning human behavior models with first-person-view camera images. Their *Darko* algorithm jointly discovers states, transitions, goals, and the reward function of the underlying *MDP* model. In another work, Vasquez et al. [180] conducted experiments to compare the performance of different *IRL* approaches, namely, *Max-margin IRL* [181] and *Maximum Entropy IRL* [182], which were later applied for robot navigation in a densely populated environment. Also, Kretzschmar et al. [183] used *Maximum Entropy IRL* to deduce the parameters of the human motion model that imitates the learned behaviors. *IRL* seeks to extract the latent reward or cost function from expert demonstrations by considering the underlying *MDP*. It learns from entire trajectories, and its computational expense arises from running *RL* in an inner loop [184]. Another approach was proposed by Goldhammer et al. [185], who used an *Artificial Neural Network* (*ANN*) with the multilayer perceptron architecture to learn usual human motion patterns. A different method was presented by Gao et al. [186], who trained a *Reinforced Encoder–Decoder* network to predict possible activities.

Alternatively, *Long Short-Term Memory* (*LSTM*) networks are one of the sequential methods that learn conditional models over time and recursively apply learned transition functions for inference [187]. Unlike standard feed-forward neural networks, also known as recurrent neural networks, these networks include feedback connections. Following the work by Alahi et al. [188], who presented a human trajectory forecasting model based on *LSTM* networks, they have become widely popular for this purpose. For example, Furnari and Farinella [189] utilized *LSTM* to predict future human actions in a domestic setting. Chen et al. [190] also created an *LSTM*-based model predicting socially aware trajectories learned from a dataset to later integrate this into a robot motion planning scheme. *Recurrent Neural Networks* (*RNN*) were also applied for sequence learning, e.g., by Vemula et al. [191] who proposed the *Social Attention* trajectory prediction model that captures the relative importance of each person when navigating in the crowd, irrespective of their proximity. Another work by Farha et al. [192] relies on training a *Convolutional Neural Network* (*CNN*) and a *RNN* to learn future sequences. They proved their method to be suited for long-term predictions of video sequences.

Another effective data-based method for learning from demonstrations is *Generative Adversarial Imitation Learning* (*GAIL*), applied by, e.g., Tai et al. [184] to learn continuous actions and desired force toward the target. Huang et al. [193] proposed a model-based interactive imitation framework combining the advantages of *GAIL*, interactive *RL*, and model-based *RL*.

On the other hand, Kanda et al. [194] used the *Support Vector Machine* (*SVM*) to classify 2 s recordings of human trajectories in a shopping mall into four behavior classes: fast walking, idle walking, wandering, and stopping. The classification relies on features of trajectory shapes and velocity. Coarse classification enables forecasting human trajectories [6]. Similarly, Xiao et al. [195] first pretrained the *SVM* to group activity classes, then predicted the trajectories based on those classes, and finally evaluated the system in a lab environment.

Alternatively, the *Social Force Model* (*SFM*) [43] with its numerous modifications [156,158,196], is also a popular method for human trajectory prediction; however, it requires knowledge about environmental cues to infer the possible goals of humans. Luber et al. [197] combined *SFM* with a tracker based on the Kalman filter to produce a more realistic prediction model of human motion under the constant velocity assumption. Recently, multiple approaches integrating *SFM* into neural network schemes were proposed. For example, Yue et al. [198] integrated *SFM* and a deep neural network in their *Neural Social Physics* model with learnable parameters. Gil and Sanfeliu [199] presented *Social Force Generative Adversarial Network* (*SoFGAN*) that uses a *GAN* and *SFM* to generate different plausible people trajectories reducing collisions in a scene.

Numerous works across various application domains depend on kinematic models for their simplicity and satisfactory performance, particularly in scenarios with minimal motion uncertainty and short prediction horizons. Among others, Elnagar [200] proposed a method predicting future poses of dynamic obstacles using a Kalman filter under the assumption of using a constant acceleration model. Similarly, Lin et al. [201] proposed a forecasting strategy that employs a bimodal extended Kalman filter to capture the dual nature of pedestrian behavior—either moving or remaining stationary. Also, Kim et al. [202] used a combination of ensemble Kalman filters and a maximum-likelihood estimation algorithm for human trajectory prediction.

In applications where performance is crucial, the constant velocity model, assuming piecewise constant velocity with white noise acceleration, can be applied. Despite its simplicity, it is commonly chosen as an ad hoc method for motion prediction in numerous approaches [139,203,204,205,206,207,208] having lightweight and straightforward implementation and yielding satisfactory results with high-frequency updates. Recently, Schöller et al. [209] discussed that the constant velocity model might outperform state-of-the-art neural methods in some scenarios.

Diverse methods were also evaluated for usage in human trajectory prediction, for example, belief distribution maps [210] that consider the obstacle situation in the robot’s environment, *multigoal Interacting Gaussian Processes* (*mgIGP*) [211] that can reason multiple goals of a human for cooperative navigation in dense crowds, or the *Human Motion Behavior Model* (*HMBM*) [212], allowing a robot to perform human-like decisions in various scenarios. Another method was proposed by Ferrer and Sanfeliu [213], who presented a geometric-based long-term *Bayesian Human Motion Intentionality Predictor* using a naive Bayes classifier that only requires training to obtain the set of salient destinations that configure a scene.

Our survey discusses the most common methods used in robotic applications, but various other methods for human trajectory prediction have evolved over the years. Rudenko et al. [187] presented a thorough review of the state-of-the-art human motion prediction methods, where they also discussed approaches that account for map information or environmental cues for predictions. An appropriate forecasting method has to be selected for a specific application based on multiple criteria, e.g., computational resources, prediction horizon, and detection uncertainty.

### 4.4. Contextual Awareness

A robot is perceived as intelligent if it utilizes the contextual information in its imperative [16,214]. The proper socially aware activity of a robot performing a single task might differ depending on the situation defined by a contextual arrangement. It is connected to adjusting the robot’s behavior, knowing what environment it is in (gallery or shopping mall), what task it performs (transporting a glass full of hot tea or packed goods), whom it interacts with (young person or elderly), and what social norms are expected in the environment (may differ between cultures).

Francis et al. [12], in their survey, identified the following forms of context: cultural context [26,34,85,215,216,217], environmental context, individuals diversity, task context, and interpersonal context, but their literature review in this area is narrow. The notion of context is usually regarded in the deliberative layer of the robot’s planning and embedded as spatial or spatiotemporal constraints in the motion planning [17,218,219].

#### 4.4.1. Environmental Context

The environmental context is constituted by various characteristics of the robot’s surroundings. This information is particularly important for robots that act in different types of rooms, e.g., corridors and libraries of the university. While the robot might be sociable and lively in corridors, it is not necessarily appropriate to distract students in the library, where the robot should move slowly and be quiet. Therefore, researchers investigate different environmental concepts to embed them into robot navigation schemes.

Banisetty et al. [220] proposed a model-based context classifier integrated with a high-level decision-making system for socially aware navigation. Their *CNN* model distinguishes between different environmental contexts such as an art gallery, hallway, vending machine, and others. Additionally, based on the *LiDAR* observations and using the *SVM*, they classified social contexts, namely people forming a queue and F-formations. In continuation of this article, Salek Shahrezaie et al. [221] introduced classification and detection information into a knowledge base they queried to extract applicable social rules associated with the context at hand. This approach has been further extended in [142] for using environmental context, object information, and more realistic interaction rules for complex social spaces. On the other hand, Jia et al. [222] proposed a deep-learning-based method for detecting hazardous objects in the environment of an autonomous cleaning robot to maintain safe distances from them on the motion planning level. Recognizing human activity spaces is a part of environmental context awareness, as presented in the work by Vega et al. [223], who exploited the detection of specific objects for this purpose.

A leading approach to enable the robot’s contextual awareness is semantic mapping [224,225,226]. For example, Zhang et al. [227] used an object semantic grid map along with a topological map for the automatic selection of roughly defined navigation goals in a multiroom scenario. Alternatively, Núñez et al. [228] proposed a navigation paradigm where the semantic knowledge of the robot’s surroundings and different social rules are used in conjunction with the geometric representation of the environment’s semantic solutions. Their approach aims to integrate semantic knowledge and geometrical information. A promising method for the interactive building of semantic maps for robot navigation is illustrated in [229].

#### 4.4.2. Interpersonal Context

Interpersonal cues are mainly related to social relationships between tracked humans in the robot environment. This knowledge can be embedded in control systems to enhance robot navigation skills. For example, Li et al. [230] proposed a dual-glance *CNN*-based model for visual recognition of social relationships. The first glance fixates on the person of interest, and the second glance deploys an attention mechanism to exploit contextual cues. Lu et al. [161] proposed an approach for context-sensitive navigation, mainly focusing on human-aware robot navigation and embedded spatial constraints into environment models in the form of costmaps.

The algorithm by Luber and Arras [168] was extended in [160] for detecting and learning sociospatial relations, which are used for creating a social network graph to track groups of humans. Patompak et al. [231] developed a *Reinforcement Learning* method of estimating a social interaction model for assisting the navigation algorithm regarding social relations between humans in the robot’s environment model. Similarly, Okal and Arras [232] employed *Bayesian Inverse Reinforcement Learning* for learning the cost function of traversing in the area with a group of humans.

Haarslev et al. [233] introduced contextual information into robot motion planning, namely F-formation spatial constraints in the costmaps used for planning. The F-formation arrangement is inferred from participants’ speed, line of sight, and potential focus points. Similarly, Schwörer et al. [234] detected people and their interactions to create spatial constraints in the environment model used for motion planning.

#### 4.4.3. Diversity Context

Diversity-related contexts facilitate leveraging human diversity in social robot navigation. Researchers presented multiple studies regarding gender [235,236,237], age [235,236,238] personality [136,239], and diverse human groups representations [240]. All these traits affect how people interact with and perceive robots. Furthermore, Bera et al. [26] attempted to classify the personality of each pedestrian in the crowd to differentiate the sizes of personal spaces of individuals. Subsequently, the emotional state of the pedestrians was also inferred and embedded for socially aware navigation [27,241,242].

#### 4.4.4. Task Context

A robot’s behavior differs based on a task to perform. If the robot is delegated to execute a task of a high priority, e.g., urgent transportation in a hospital, it will interact with humans only in an unfocused manner committing to collision avoidance and respecting personal spaces. However, if the robot’s task is to start sociably interacting with customers in a shopping mall to present products to them, it has to mildly start focused interactions with pedestrians. Therefore, the objectives of robot navigation differ between tasks, affecting the socially correct behavior scheme that should be followed.

Popular tasks delegated to social and assistive robots are transportation [79], guiding [160,243], or accompanying [157,244]. For example, accompanying objectives differ even between the tasks of attending individuals [244,245] and groups [157,246] or even between different strategies for accompanying individuals (Section 3.5.1). Similarly, a guiding robot, e.g., proposed in [243], mainly focuses on leader–follower tasks, but once it finishes the guided tour, it may drop the constraints specific to the guiding behavior (speed, etc.) and switch to socially aware collision avoidance and back to the reception area.

A significant challenge lies in integrating the contradictory objectives of treating humans as social obstacles during tasks requiring only unfocused interactions and regarding them as interaction partners when needed. As a result, methods introducing human awareness and social acceptance must be carefully selected to avoid interfering with contradictory modes of operation, as some constraints may need to be disabled in focused interaction mode while enabled in unfocused interaction mode [23].

## 5. Motion Planning

Robots using socially aware navigation planners are perceived to be more socially intelligent than those using traditional navigation planners as studied in [247]. This section discusses various navigation approaches and methods of incorporating social awareness into robot control systems.

The motion planning module is crucial for safely guiding the robot through dynamic environments. Motion planning for mobile robots is understood as a pose control scheme aimed at moving the robot from its initial pose to the target pose while considering the kinematic and dynamic (kinodynamic) constraints of the mobile base.

From the perspective of motion planning, requirements for social awareness presented in Section 3 might entail the necessity of specific enhancements compared with classical robot navigation. Namely, those can be classified into three specific groups. Firstly, modifications of the intermediate trajectory to the fixed goal. This might involve adjustments originating from respecting personal spaces (**Req. 2.1**), O-spaces of F-formations (**Req. 2.2**), and modulating speed (**Req. 2.3**) to mitigate the discomfort of surrounding humans. Secondly, the extended selection of the final poses for navigation tasks with coarsely defined goals. In particular, selecting such a pose that, e.g., does not block any affordance space (**Req. 4.2**), minimizes the discomfort of the approach to a human (**Req. 2.5.1**), or provides joining a queue in a socially compliant manner (**Req. 4.6**). Thirdly, dynamically inferring and following virtual goals in real time depending on the poses of cooperating humans, which enables efficient execution of accompanying tasks (**Req. 4.1**).

The predominant motion planning architecture for mobile robots relies on hierarchical planning with two asynchronously running modules, specifically, a global path planner and a local trajectory planner [138,248]. Global path planning involves finding a feasible path from a start configuration to a goal configuration while avoiding environmental obstacles. Algorithms generating global paths typically operate in a configuration space and consider the entire environment [249]. In contrast, local trajectory planning aims to generate trajectories for the robot to follow within a short time horizon that navigate the robot safely and efficiently through the environment while reacting to dynamic obstacles and perturbations. Algorithms producing local trajectories typically operate in the robot’s control space or velocity space and consider immediate sensor feedback and environmental information [138,152]. Usually, local trajectory planners operate at a higher frequency than global path planners to adjust the robot’s motion in real time, accounting for dynamic changes in the environment and ensuring safe and efficient navigation.

**Figure 8:**
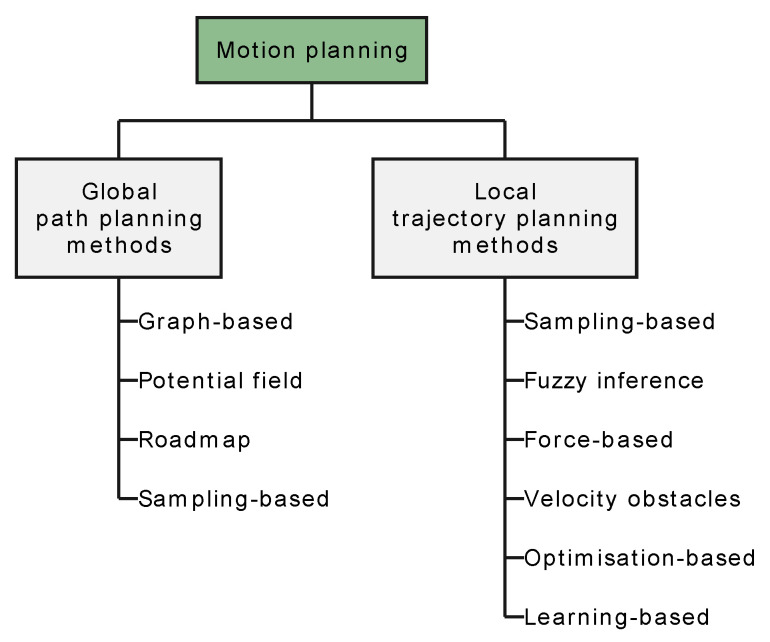
A taxonomy of motion planning for social robot navigation.

Our taxonomy of the algorithmic perspective of social robot navigation follows the hierarchical motion planning scheme, differentiating approaches for global path planning and local trajectory planning Figure 8.

Numerous surveys regarding social robot navigation thoroughly discussed motion planning [13,14,15]. However, our review aims not only to investigate the variety of methods of implementing human awareness in robot control systems but also to classify those approaches according to the requirements they fulfill. The classification of requirements regarded in objectives of different navigation algorithms is presented in Section 5.3.

### 5.1. Global Path Planning

In the context of global path planning for social navigation for surface robots, various methodologies are employed for the research. Recently, multiple surveys regarding path planning for mobile robots have been proposed [250,251,252,253,254]. State-of-the-art techniques can be classified into distinct groups. These include graph-based methods, potential field methods, roadmap methods, and sampling-based methods. Each class of approaches offers unique advantages and challenges, contributing to the broader landscape of mobile robot path planning.

Although in classical path-planning metaheuristic methods like genetic algorithms or particle swarm optimization are commonly discussed [255], to the best of our knowledge, they were not applied for human-aware navigation.

#### 5.1.1. Graph-Based Methods

Graph-based methods for path finding fall into the category of approximate cell decomposition approach in which cells of predefined shape (usually rectangles) do not exactly cover the free space (in contrast to exact cell decomposition), but the cell connectivity in a graph is encoded [256].

##### Algorithms

The earliest graph (or grid) search methods in the context of computer science and algorithmic development can be traced back to the 1950s. One significant development was Dijkstra’s algorithm [257], which laid the foundation for many subsequent graph search and pathfinding algorithms. This algorithm was primarily focused on finding the shortest path in a graph. Later, Hart et al. [258] presented the $A^{*}$ algorithm, which builds upon Dijkstra’s algorithm by incorporating heuristic information to guide the search more efficiently, making it particularly useful for pathfinding in large graphs. The heuristic utilizes the distance between the current processing node and the goal node on the solution space. Globally shortest paths are obtained using both heuristic estimates and actual costs in a weighted graph. Other variants of the $A^{*}$ planning algorithm include $D^{*}$ [259], *Focused* $D^{*}$ [260], ${LPA}^{*}$ [261], $D^{*}$ *Lite* [262], $E^{*}$ [263], *Field* $D^{*}$ [151], and ${Theta}^{*}$ [264]. A brief description of each variant is depicted below.

Graph-based planners usually require replanning if the underlying environment model changes. This drawback is addressed by the $D^{*}$ [259], which is an incremental search algorithm for finding the shortest paths designated particularly for graphs that may dynamically change once the search begins as it possesses the procedure for updating paths if changes occur. *Focused* $D^{*}$ [260] adapts the $D^{*}$ to prioritize the exploration of areas closer to the goal. *Lifelong Planning* $A^{*}$ (${LPA}^{*}$) [261] is an incremental heuristic search algorithm that continuously improves its estimates of the shortest path while adapting to changes in the environment, providing efficient planning in dynamic environments. $D^{*}$ *Lite* [262] is a simplified version of the $D^{*}$ algorithm, focusing on efficient replanning for real-time performance in static or partially unknown environments. The wavefront expansion procedure (known as *NF1* in [256]) is a simple global planner that expands the search to all adjacent nodes until the start node and goal node are covered. It was employed in [212] for path planning in human-populated environments. Another method is $E^{*}$ [263] algorithm capable of dynamic replanning and user-configurable path cost interpolation. It calculates a navigation function as a sampling of an underlying smooth goal distance that takes into account a continuous notion of risk that can be controlled in a fine-grained manner.

The authors of *Field* $D^{*}$ [151] addressed the problem of using discrete state transitions that constrain an agent’s motion to a narrow set of possible headings, which often occurs in classical grid-based path planners. Instead, they proposed the linear interpolation approach during planning to produce paths with a continuous range of headings. Alternatively, the ${Theta}^{*}$ [264] method propagates information along grid edges (to achieve a short runtime) but without constraining the paths to the grid edges. Instead, *any-angle* paths are found by performing line-of-sight checks between nodes. When a direct line of sight is feasible between two adjacent nodes without intersecting obstacles, ${Theta}^{*}$ considers the straight-line path, reducing the number of nodes expanded, compared with $A^{*}$. Also, ${Theta}^{*}$ retains the optimality guarantees of $A^{*}$ while producing smoother, more natural paths, especially in environments with narrow passages or obstacles.

Notably, Dijkstra’s algorithm does not account for the robot’s kinodynamic constraints, which may generate paths not admissible to robots with, e.g., Ackermann kinematics. However, Dolgov et al. [265] addressed this issue in their *Hybrid* $A^{*}$ algorithm that extends the traditional $A^{*}$ to handle continuous state spaces by discretizing them into a grid. It incorporates vehicle kinematic constraints, such as maximum velocity and steering angle, to generate feasible paths for vehicles navigating through complex environments. Recently, Macenski et al. [249] presented a search-based planning framework with multiple algorithm implementations, including the Cost-Aware Hybrid-A* planner that provides feasible paths using a Dubins or Reeds–Shepp motion model constrained by a minimum turning radius for Ackermann vehicles.

##### Human-Aware Constraints

The classical path-finding algorithms focus on calculating the shortest, collision-free path and do not explicitly regard humans in the environment; hence, they also do not consider social constraints. However, in graph-based methods, the planning procedure is separated from the definition of planning constraints incorporated into the environment representation [206]. Hence, researchers started to modify the environment models, e.g., costmaps, to embed human-aware constraints into the motion planning scheme while employing classical path-finding algorithms. Most approaches that extend environment representations focus on introducing spatial or spatiotemporal soft constraints representing proxemics [266] or social conventions [59,161].

For example, Sisbot et al. [266] presented a *Human Aware Motion Planner* (*HAMP*) that exploits algorithms for reasoning on humans’ positions, fields of view, and postures. They integrated different social constraints into their highly configurable planning scheme, including Gaussian-modeled personal spaces or hidden zones behind obstacles (visibility constraints). Kirby et al. [59] proposed a *Constraint-Optimizing Method for Person-Acceptable NavigatION* (*COMPANION*) framework in which, at the global path-planning level, multiple human social conventions, such as personal spaces and tending to one side of hallways, are represented as constraints on the robot’s navigation.

Lu et al. [73] presented a costmap-based system capable of creating more efficient corridor navigation behaviors by manipulating existing navigation algorithms and introducing social cues. They extended robot environment models with socially aware spatial constraints to navigate in a more human-friendly manner. Kollmitz et al. [206] presented a planning-based approach that uses predicted human trajectories and a social cost function to plan collision-free paths taking human comfort into account. They employed search-based, time-dependent path planning that accounts for the kinematic and dynamic constraints of a robot. The authors also exploited the layered costmap architecture [161] to create multiple layers related to human proxemics according to their prediction model. Okal et al. [232] proposed a method that uses *IRL* to learn features of a populated environment to model socially normative behaviors [180]. Once the reward function for a navigation task is obtained, it is used to define spatial costs of social normativeness that can be injected into a costmap used by a motion planner (either global or local). Some works also embedded dynamically recalculated personal zones into costmaps to account for dynamics of individual humans [59,244,267,268] or groups [269].

#### 5.1.2. Potential Field Methods

Purely graph-based planners have limitations originating from their discontinuous representation of configuration space. On the other hand, potential field methods offer smoother path generation and can be directly related to sensor data, yet they suffer from the presence of local minima [263]. Path planning utilizing a potential field creates a gradient across the robot’s map that directs the robot to the goal position from multiple prior positions [256].

One of the pioneering works that introduced the concept of *Artificial Potential Field* (*APF*) for obstacle avoidance and navigation in robotics is [91]. The potential field methods treat the robot as a point in the configuration space under the influence of an *APF*. The goal, acting as a minimum in this space, exerts an attractive force on the robot, while obstacles act as repulsive forces. The superposition of all forces is applied to the robot. Such an *APF* smoothly guides the robot toward the goal while simultaneously avoiding known obstacles, just as a ball would roll downhill [270].

Later, Borenstein and Koren [271] developed a *Virtual Force Field* method that relies on two basic concepts: certainty grids for obstacle representation and potential fields for navigation. Their method enables continuous motion of the robot without stopping in front of obstacles with a speed of 0.78 m/s. However, the approach was abandoned due to the method’s instability and inability to pass through narrow passages [270]. The extended potential field method has been proposed by Khatib and Chatila [272] with two additions to the basic potential field, in particular, the rotation potential field and the task potential field.

More recently, Iizuka et al. [273] proposed a modified *APF* approach resistant to the local minimum issue in multiobstacle environments, while Weerakoon et al. [274] presented a deadlock-free *APF*-based path-planning algorithm. Similarly, Azzabi and Nouri [275] developed an approach that addresses the common issues of the original *APF*, namely local minima and the goal being nonreachable with obstacles nearby. Szczepanski [276] also proposed a path-planning method for mobile robots that uses the attractive potential for goal reaching as the original *APF*, but the repulsive potential is replaced by a general obstacle potential, equal to repulsive potential, vortex potential, or their superposition.

#### 5.1.3. Roadmap Methods

Roadmap strategies capture the connectivity of the robot’s unobstructed space through a network of 1D curves or lines, denoted as roadmaps. Subsequently, the roadmap serves as a network of path segments for planning robot movement. Consequently, path planning is reduced to connecting the robot’s initial and goal positions to the road network, followed by identifying a sequence of routes from the initial robot position to its destination [270]. The most common approaches falling into the roadmap-based category are visibility graphs and Voronoi diagrams.

The visibility graph method is one of the earliest path-planning methods [256]. For a polygonal configuration space, the graph consists of edges joining all pairs of vertices that can see each other (including both the initial and goal positions as vertices as well). The unobstructed straight lines (roads) joining those vertices are the shortest distances between them, guaranteeing optimality in terms of the length of the solution path. The main caveat of the visibility graph is that the solution paths tend to move the robot as close as possible to obstacles on the way to the goal [270]. In contrast, the Voronoi diagram is an approach that maximizes the distance between the robot and obstacles in the map [270].

Our research regarding the applications of classical roadmap methods shows that they are rarely used in social robot navigation as they only consider binary environment models (obstacle or free space); hence, human awareness cannot be properly tackled. However, Voronoi diagrams might be used as reference path-planning approaches [204,277,278,279] for capturing the skeleton of the environment along with human-aware trajectory planners as in [132].

#### 5.1.4. Sampling-Based Methods

The main idea of sampling-based motion planning is to avoid the explicit construction of obstacle regions but instead conduct a search that probes the configuration space with a sampling scheme [280]. The most prevalent methods falling into the category of sampling-based path planners are the Probabilistic Roadmap (*PRM*) [281] and the Rapidly exploring Random Trees (*RRT*) [282], both being probabilistically complete [280].

##### Algorithms

*PRM* [281] constructs a roadmap, a graph representation of the configuration space, by sampling random points and connecting them with collision-free paths. It focuses on building a network of feasible paths between different regions of the configuration space and is effective for multiquery scenarios or environments with complex obstacles.

*RRT* [282] builds a tree structure by iteratively selecting random points in the configuration space and extending the tree towards those points. It explores the configuration space rapidly and is particularly effective for high-dimensional spaces. Different variants of *RRT* have been developed, including *RRT-Connect* [283], ${RRT}^{*}$ [284] or dual-tree version—*DT-RRT* [285].

Both *PRM* and *RRT* have different characteristics. *PRM* requires a two-phase process: first, constructing the roadmap offline and then querying the roadmap online to find a path between a start and goal configuration. In contrast, *RRT* performs exploration and path planning simultaneously, gradually growing towards the goal configuration during the search process. *PRM* is a well-suited method for scenarios where the environment is relatively static and the planner has sufficient computational resources to construct the roadmap offline, while *RRT* is often favored for real-time or dynamic environments, as it can adaptively explore the space and find feasible paths in a run-time. A notable feature of sampling-based methods is that these planners can regard the kinodynamic limits of the robot to generate feasible and safe motion plans in continuous state and action spaces.

##### Human-Aware Constraints

Some works focus on including constraints related to social conventions in sampling-based path-planning schemes. For example, Svenstrup et al. [286] modified the original *RRT* for navigation in human environments assuming access to full state information. Their modifications include adding the potential model designed for moving humans, so the customized *RRT* planner plans with a potential field representation of the world. Similarly, Rios-Martinez et al. [287] proposed *Risk-RRT* for global path planning. Their algorithm includes the knowledge of the personal spaces of pedestrians and the possible interactions between the F-formation’s participants. *Risk-RRT* penalizes the robot’s crossing through personal spaces and O-spaces of F-formations by assigning additional costs to those areas. Furthermore, Shrestha et al. [288] used *RRT* for global path planning in the environment with a stationary human. Vega et al. [223] attempted to integrate proxemics theory with their path planner incorporating *PRM* [289] and *RRT* [282] methods by defining personal spaces and activity spaces as forbidden areas for robot navigation. Alternatively, Pérez-Higueras et al. [290] developed a cost function for the *RRT*-based path planner employing *Inverse Reinforcement Learning* from demonstrations.

### 5.2. Local Trajectory Planning

The most common architecture for robot motion planning separates global path planning and local trajectory planning [138,248]. This separation of concerns allows for modular and flexible robotic systems, where different strategies can be applied at each level of abstraction to address specific requirements.

Local trajectory planners generate trajectories for the robot to follow within a short time horizon. Short time horizons allow operating with a higher frequency to instantly react to environmental changes and possible encounters. Trajectory planners operate in the robot’s control space or velocity space and regard not only spatial aspects of motion planning but also temporal ones. In the following part of this survey, various trajectory planning methods and approaches to incorporating human awareness into robot behavior are reviewed.

#### 5.2.1. Sampling-Based Methods

Besides global path planning Section 5.1.4, sampling-based methods can also be applied to local trajectory planning. An extended *RRT* with a notion of time included—spatiotemporal *RRT*—was proposed by Sakahara et al. [204]. Their method integrates ideas of the *RRT* and the Voronoi diagram. Although motion prediction of dynamic objects is regarded, they do not explicitly capture social conventions. Nishitani et al. [205] extended this approach, presenting a human-centered *X–Y–T* space motion planning method. The authors included human personal space and directional area as well as the robot’s dynamic constraints in the planning scheme.

Pérez-Higueras et al. pointed out in [291] the future work perspective of using *RRT* as a local trajectory planner due to real-time capability, but their further work leaned toward learning-based approaches.

#### 5.2.2. Fuzzy Inference Methods

Fuzzy inference systems (*FIS*) form another well-established paradigm for control systems, specifically useful to model imprecise or non-numerical information and decisions. *FIS* are applied for traditional robot navigation [292,293,294,295,296] and social robot navigation tasks [297,298,299,300]. They can also be integrated with other approaches, e.g., *Q-learning* [301] or *Reinforcement Learning* [302].

An example of the *FIS* method adapted for human-aware robot navigation is the work by Palm et al. [297], who derived fuzzy control rules for the robot’s actions based on expected human movements relative to the robot. They investigated the movement of humans in a shared space with a robot to determine lane preference and agent classification for collision avoidance. Another method was proposed by Obo and Yasuda [298], who developed a framework for robot navigation in crowds employing multiobjective behavior coordination for collision avoidance. Rifqi et al. [299] used *FIS* to dynamically change parameters of the *SFM*, which has been applied for controlling the movement of a healthcare robot. Rules that they designed switch the robot’s motion behavior based on its distance to human proxemics zones. Recently, Sampathkumar et al. [300] proposed a framework integrating an *Artificial Potential Field* and *FIS* for navigation that prioritizes safety and human comfort.

#### 5.2.3. Force-Based Methods

Force-based approaches model the motion of individuals (humans or robots) in the environment considering the forces acting on them. These include a force attracting the agent to the goal and forces arising from interactions with other agents and environment objects such as obstacles. Typically, they are purely reactive methods that decide the next movement based on the environment arrangement at hand, i.e., obstacles and human locations. The resultant force can be directly transformed into a velocity command for a robot. The predominant methodologies within this category are *Elastic Bands* [303] and *Social Force Model* [43].

*Elastic Bands* [303] is a method that aims to close the gap between global path planning and reactive control, as it performs local path deformation based on internal and external forces. Internal forces contract the path, favoring the shortest path to the goal, while external forces repel the path from obstacles. The authors of the algorithm proposed a reference implementation based on bubbles that represent discrete path points and free space. Later, this method was extended by Brock et al. [304] mainly for motion generation in manipulation tasks performed in human environments. More recently, a socially aware specialization focusing on improving motion legibility of the *Elastic Bands* local trajectory planner has been developed for the SPENCER project [160]. The notion of human awareness has also been implemented into the *Elastic Bands* approach by Vega et al. [223].

On the other hand, *Social Force Model* (*SFM*) [43] has been one of the prevalent methods for crowd behavior simulation [305,306], human trajectory prediction (Section 4.3), and human-like motion generation in robotics. It constitutes a model inspired by fluid dynamics that illustrates an agent’s motion using a set of attractive and repulsive forces. Its flexible formulation allows for capturing additional models of social phenomena to obtain more realistic motion behaviors. Therefore, the original approach has undergone multiple extensions and over the years numerous successful real-world robotic applications have emerged [9,156,157,158,245,307,308].

Researchers expanded the basic *SFM* with explicit collision prediction [196,309], making the behavior more proactive and anticipatory. Kivrak et al. [158] also introduced collision prediction into *SFM*-based model which they integrated with a robot operating in an unknown environment with no a priori map. Similarly, Shiomi et al. [9] evaluated *SFM* with collision prediction [196] in a real-world shopping mall. Collective motion conventions were also integrated into the model formulation [310], as well as group formations [61,311,312]. Some works also focused on improving the realism of generated trajectories [313].

Truong and Ngo [307] proposed a proactive social motion model for safe and socially aware navigation in crowded environments. Their formulation takes into account the socio-spatiotemporal characteristics of humans, including human body pose, field of view, hand poses, and social interactions, which consist of human–object interaction and human group interaction.

Furthermore, Ferrer et al. [308] presented another model that extends the original formulation to effectively accompany a person. They implemented human behavior prediction to estimate the destination of the person the robot is walking with. Additionally, the authors exploited the parameterization of the *SFM* and applied a method of interactively learning the parameters of the model using multimodal human feedback.

Moreover, Repiso et al. presented studies regarding the robot accompanying single humans [245] and human groups [157]. In [245], they implemented three stages of focused interaction between the robot and a human: accompanying, approaching, and positioning. They inferred the human’s final destination (among all destinations marked in the environment beforehand) and predicted the human motion with the *SFM*. The *SFM* was also employed for the robot’s local trajectory planning, and spatial cost functions were used for trajectory scoring. In the following work, Repiso et al. [157] proposed an extended method that allows the robot to break the ideal side-by-side formation to avoid other people and obstacles, implementing the human-aware robot navigation strategy for accompanying groups of multiple humans.

Alternatively, Ferrer and Sanfeliu [156] developed a *SFM*-based *Anticipative Kinodynamic Planning* method for unfocused interactions between a robot and humans. They implemented a scalarised multiobjective cost function to choose the best trajectory amid the generated ones. On the other hand, We et al. [314] proposed a pedestrian’s heterogeneity-based social force model that captures the physiology and psychology attributes of pedestrians introducing physique and mentality coefficients into the *SFM*. Recently, *SFM* has also been involved in approaches integrating machine learning techniques with motion models [199,315].

#### 5.2.4. Velocity Obstacles Methods

The *Velocity Obstacle* (*VO*) [316] concept is a foundation for a broad class of proactive methods for a robot’s local navigation. *VO* methods are based on a persistent effort to keep a robot collision-free, requiring only: a radius, a position, and a speed of each robot [317]. They generate avoidance maneuvers by selecting the robot velocities outside the collision cone, which consists of velocities that in the future would result in close encounters with obstacles moving at known velocities. A practical application of *VO* was introduced by Lin et al. [318]. They adapted the concept by assuming that each agent is a decision-making entity capable of selecting the appropriate velocity that responds to the other agents’ movements and replanning its path. Moreover, an extension of *VO*, called *Reciprocal Velocity Obstacle* (*RVO*), was developed by van den Berg et al. [319]. They exploited the fact that humans in the environment cooperate [320] and the approach guarantees to generate safe and oscillation-free motions under an assumption that all dynamic agents make a similar collision-avoidance reasoning [14]. Furthermore, a related method called *Optimal Reciprocal Collision Avoidance* (*ORCA*) [321] does not require implicit communication between agents and optimizes global objectives when finding collision-free velocities.

*VO*-based methods are rarely enhanced with socially aware concepts. Martinez-Baselga et al. [143] presented a *Strategy-based Dynamic Object Velocity Space* trajectory planner that explicitly regards the presence of dynamic obstacles but does not take any social conventions into account. Similarly, Zhang et al. [139] proposed a local trajectory planning scheme using *ORCA* that includes uncertainties of states of surrounding humans when selecting collision-free velocities.

#### 5.2.5. Optimization-Based Methods

Another class of approaches for human-aware trajectory planning formulates the problem as an optimization task, which relies on finding control inputs that optimize (minimize or maximize) an objective function while satisfying kinodynamic and collision-free motion constraints. These hard constraints, inherited from classical robot navigation, restrict control inputs to those feasible for the specific mobile base at a given time and ensure the absence of collisions within the prediction horizon. The presence of collisions with the surrounding objects is assessed using the environment model and forward simulation of applying the computed controls. In contrast, soft constraints are embedded in the optimized objective function that takes into account, e.g., intrusions into the personal spaces of humans.

Most state-of-the-art methods planning optimal socially aware local trajectories extend the classical robot navigation algorithms, namely *Dynamic Window Approach* (*DWA*) [135] and *Timed Elastic Bands* (*TEB*) [153].

##### *DWA*-Based Methods

The *DWA* is one of the most common algorithms for collision avoidance. The main characteristic of the approach is that commands, controlling the translational and rotational velocities of the robot, are searched directly in the space of velocities. The search space is reduced to velocity pairs fulfilling kinodynamic constraints. Typically, for each velocity pair, the effect of applying those controls to the robot is simulated over a short time horizon, e.g., 1.5–3.0 s, which produces multiple circular trajectories. The optimal trajectory is the one maximizing the objective function consisting of three weighted components. In particular, the components evaluate the progress toward the goal, the distance to the closest obstacle, and the forward velocity of the robot. Numerous modifications of *DWA* have been proposed, as the objective function is expandable [322,323]. However, the method does not explicitly capture the dynamics of the obstacles taking into account only their current position.

Another method, *Trajectory Rollout* [152] is similar to *DWA* but exhibits one essential difference—in each forward simulation step, a set of feasible velocity pairs is updated as the kinematic constraints are recalculated according to the current velocity and dynamic constraints.

Constraints related to social conventions are usually embedded in the environment representation used by trajectory planners [210] or by extending the objective function [212,324]. For example, Weinrich et al. [210] applied the $E^{*}$ algorithm as a global path planner along with an extended *DWA* method as a local trajectory planner. They extended *DWA* with an additional objective rating that considers spatiotemporal occupation probabilities of the tracked humans. In particular, they assigned personal spaces to humans using *Gaussian Mixtures*. The method provided successful collision avoidance by the robot in a passing scenario of a narrow hallway. A similar extension of *DWA* was proposed in [325].

Seder et al. [324] and Oli et al. [212] proposed navigation approaches that employed a modified *DWA* for human-aware local trajectory planning. They introduced human awareness by modifying the objective component related to clearance from obstacles, in particular, including predicted poses of tracked humans as future obstacle positions. The difference between these methods is that in [324] the authors assumed human motion predictions driven by the constant velocity model, while in [212] the *SFM* has been implemented. Also, the method from [324] used *Focused* $D^{*}$ as a global path planner, whereas in [212], the *NF1* [256] was integrated.

##### *TEB*-Based Methods

The *TEB* is a traditional local trajectory planner that laid a foundation for multiple methods that enhanced this approach to capture human-awareness constraints [159,207,326]. The basic *TEB* deforms local trajectories according to the locations of obstacles in the environment, but, in contrast to *Elastic Bands*, with temporal information. Instead of forces from *Elastic Bands*, *TEB* uses an optimization objective to follow the global path regarding kinodynamic constraints, forming the optimization problem of nonlinear least squares.

Human-aware specialization of *TEB*, named *HaTEB*, was proposed by Khambhaita and Alami [207]. They extended the original optimization constraints with safety (minimum safety distance), time to collision, and directional constraints, including the predicted human trajectories in the problem formulation. Singamaneni et al. [159,208] developed the *CoHAN* planner—the *HaTEB* extension that handles large numbers of people and focuses on motion legibility improvements. The *CoHAN* has different tunable planning modes that can handle various indoor and crowded scenarios. Recently, Hoang et al. [326] presented *GTEB* model that extends *TEB* taking into account the robot’s current state, robot dynamics, dynamic social zones [267], regular obstacles, and potential approaching poses to generate the socially optimal robot trajectory.

##### Other Methods

Alternatively to *DWA*- and *TEB*-based methods, Forer et al. [327] proposed the *Pareto Concavity Elimination Transformation* (*PaCcET*) local trajectory planner. It aims to capture the nonlinear human navigation behavior, scoring trajectories with multiple objectives. The first relies on path distance, goal distance, heading difference, and distance to obstacles, while the second is based on the interpersonal distance between the robot and humans. Later, Banisetty et al. [220] extended *PaCcET* with social awareness objectives, specifically, maintaining appropriate distances to F-formations (groups) and distance to a scenario-dependent social goal.

In contrast, the authors of [328] proposed a planner that aims to exaggerate motions to increase intent expressiveness over passing sides for legible robot navigation [72]. They implemented a decision-making strategy, constructing the *Social Momentum* objective that takes pairwise momentum between robot and human into consideration. Another method was presented by Mehta et al. [329] who applied *MultiPolicy Decision Making* to navigate in dynamic environments with different policies, namely, *Go-Solo*, *Follow-other*, and *Stop*. The values of utility functions, which compromise between the distance traveled to the goal and the disturbance to surrounding agents caused by the robot, are predicted through forward simulation.

Optimal control techniques have also been employed to maintain the formation integrity [330,331]. For instance, in [330], formation control in a leader-follower arrangement was discussed. The authors developed a method that, under mild assumptions, guarantees the stabilization of the formation to the desired shape and scale. Similarly, an optimal control algorithm, but for sustaining formations of various structures, was proposed in [331]. On the other hand, Truc et al. [332] developed a 3D reactive planner for human-aware drone navigation in populated environments that is based on a stochastic optimization of discomfort caused by the drone’s proximity to pedestrians and the visibility of the drone.

#### 5.2.6. Learning-Based Methods

In recent years, rapid growth in the machine learning field has been observed, and numerous planning approaches have evolved to capture the intricacies of human behaviors and transfer them into robot control strategies. The broadest attention in robot control applications gained *Reinforcement Learning* (*RL*) and *Deep Reinforcement Learning* (*DRL*). Specialized surveys on the applications of *RL* methods for robot navigation [333] and particularly on social robot navigation have already been published [334].

##### *Inverse Reinforcement Learning* 

A distinctively useful method for learning from demonstration is *Inverse Reinforcement Learning* (*IRL*) [181], as it allows to model the factors that motivate people’s actions instead of the actions themselves [180]. Example applications of *IRL* methods for human motion prediction were already presented in Section 4.3, but they might also be used for control purposes. For example, Kim and Pineau [335] learned a cost function involving social cues from features extracted from the *RGB-D* camera. Their *IRL* module uses a set of demonstration trajectories to learn the reference behavior when faced with different state features. Their approach is implemented as a trajectory planner with *IRL*-based cost function operating along with a global path planner. Similarly, Kuderer et al. [336] also use *IRL* with human demonstrations, but they extract features from the human trajectories and then use entropy maximization to determine the robot’s behavior during navigation in human environments. Pérez-Higueras et al. [291] also used *IRL* to transfer human motion behavior to a mobile robot. They evaluated different *Markov Decision Process* models and compared them with the baseline implementation of a global path planner and local trajectory planner without social costs. More recently, Karnan et al. [337] collected a large-scale dataset of socially compliant navigation demonstrations. They used it to perform behavior cloning [338] for a global path planner and local trajectory planner agents that aimed to mimic human navigation behaviors. The authors also performed an evaluation study for the learned approach, comparing it with a baseline *ROS* implementation.

##### *Reinforcement Learning* 

In contrast to *IRL*, the *RL* is used when the reward function is known or can be easily defined, and the goal is to find the best policy for achieving cumulative rewards. Recent works present the *DRL* as a framework to model complex interactions and cooperation, e.g., in social robot navigation.

In a study by Olivier et al. [320], the authors found that walking people mutually adjust their trajectories to avoid collision. This concept was exploited by Silva and Fraichard [339], whose approach relies on sharing motion effort between a robot and a human to avoid collisions. They learned a robot behavior using the *RL* to solve the reciprocal collision avoidance problem during simulated trials.

Li et al. [174] presented a *Role Playing Learning* formulated under a *RL* framework for purely local navigation of a robot accompanying a pedestrian. In their approach, the robot takes into account the motion of its companion to maintain a sense of affinity when they are traveling together towards a certain goal. A navigation policy is trained by *Trust Region Policy Optimization* with the use of features extracted from a *LiDAR* along with the goal as an input to output continuous velocity commands for navigation.

A series of works by Chen et al. [340,341] developed *Collision Avoidance with Deep Reinforcement Learning* (*CADRL*) approaches. Specifically, in a *Socially Aware CADRL* (*SA-CADRL*) [341], they designed a hand-crafted reward function that incorporates the social convention of passing side and enables a robot to move at human walking speed in a real-world populated environment. Everett et al. [154] proposed a *GPU/CPU Asynchronous Advantage Actor-Critic CADRL* (*GA3C-CADRL*) strategy that employs *LSTM* to use observations of arbitrary number or surrounding agents, while previous methods had this size fixed. A distinctive characteristic is that their algorithm learns collision avoidance among various types of dynamic agents without assuming they follow any particular behavior rules.

Jin et al. [342] presented another *DRL* method but for mapless collision-avoidance navigation where humans are detected using *LiDAR* scans. The reward function regards ego-safety, assessed from the robot’s perspective, and social safety, which evaluates the impact of the robot’s actions on nearby humans. The ego-safety zone maintains 0.4 m of separation between the robot and other objects, while social safety aims to prevent intrusions into approximated human personal space. Liang et al. [146] developed a *RL*-based collision-avoidance algorithm, named *CrowdSteer*, for navigation in crowded environments. The authors trained the algorithm using *Proximal Policy Optimization* (*PPO*) in high-fidelity simulation and deployed the approach on two differential drive robots.

Chen et al. [343] discussed extending pairwise interactions between the robot and individual humans to a robot interacting with a crowd. The authors developed *Socially Attentive Reinforcement Learning* (*SARL*) that jointly models human–robot as well as human–human interactions in an attention-based *DRL* framework by learning the collective importance of neighboring humans with respect to their future states. Their work was further enhanced by Li et al. [344] who addressed the problems of learned policies being limited to certain distances associated with the training procedure and the simplified environment representation that neglects obstacles different from humans. In their ${SARL}^{*}$ method, they introduced a dynamic local goal-setting mechanism and a map-based safe action space.

Guldenring et al. [345] proposed another *DRL*-based system to train neural network policies for local trajectory planning explicitly taking nearby humans into consideration. The approach uses *Proximal Policy Optimization* (*PPO*) as the main learning method while *DRL* agents are trained in randomized virtual 2D environments interacting with humans in an unfocused manner for plain collision avoidance.

Recently, Xie and Dames [147] proposed *DRL* policy for robot navigation through obstacle-filled and populated areas that intend to be generalizable to new environments. In particular, the *DRL-VO* reward function contains a novel term based on *VO* (Section 5.2.4) to guide the robot to actively avoid pedestrians and move toward its goal. In turn, Qin et al. [346] introduced a socially aware robot mapless navigation algorithm employing *RL* to learn strategies that conform to social customs and obey specific traffic rules.

##### Miscellaneous Approaches

Besides the aforementioned methods, learning-based applications include employing *Hidden Markov Model* (*HMM*) in a higher hierarchy system to learn choosing between the *RL*-based collision avoidance and target pursuing [347].

On the other hand, Tai et al. [184] attempted to apply *Generative Adversarial Imitation Learning* (*GAIL*) strategy to navigate in populated dynamic environments in a socially compliant manner via only raw depth inputs from *RGB-D* camera. Their approach learns continuous actions and desired force toward the target and outperformed pure behavior cloning policy regarding safety and efficiency.

In the approach by Lu et al. [348], the crowd’s density is dynamically quantified and incorporated into a reward function deciding the robot’s distance from pedestrians. The authors extended the *DRL*-based work from [343] so the best action is inferred from a reward function that regards the uncomfortable distance between the robot and a human. Alternatively, a system proposed by Yao et al. [114] incorporates a *Generative Adversarial Network* to track and follow social groups.

### 5.3. Discussion

A summary of discussed navigation methods according to the requirements they implement is presented in Table 2. The approaches listed in most cases employ the hierarchical structure in the motion planning system composed of a global path planner and a local trajectory planner. However, not all works explicitly reveal the planning algorithms used; thus, we do not show the details in that matter.

Each reviewed navigation method is classified based on the objectives addressed in the approach. However, the consequence of this methodology is that behavior cloning or imitation learning Section 5.2.6 are excluded from this classification, as without investigating the dataset, it is not clear which features were captured; hence, which requirements were targeted. On the other hand, *VO*-based methods (Section 5.2.4), which proactively adjust motion direction to avoid collisions, are always denoted as respecting *motion legibility* (**Req. 2.4**) (Section 3.3.4).

The requirements group most covered is by far the *physical safety* (**Req. 1**) inherited by social robot navigation from traditional navigation. It regards collision avoidance; hence, even approaches that do not explicitly regard humans in the environment (but rather moving obstacles) fall into this category. The most popular objective among social robot navigation algorithms is respecting personal spaces. However, in most methods, they are modeled as a circular shape, while many studies revealed their asymmetry (Section 3.3.1). In contrast, *motion naturalness* and, importantly, *social conventions* aspects, are less frequently discussed. The latter are rarely considered, since in research robots are usually designated for specific tasks, which influences a fragmentary approach to design and implementation.

## 6. Evaluation

**Figure 9:**
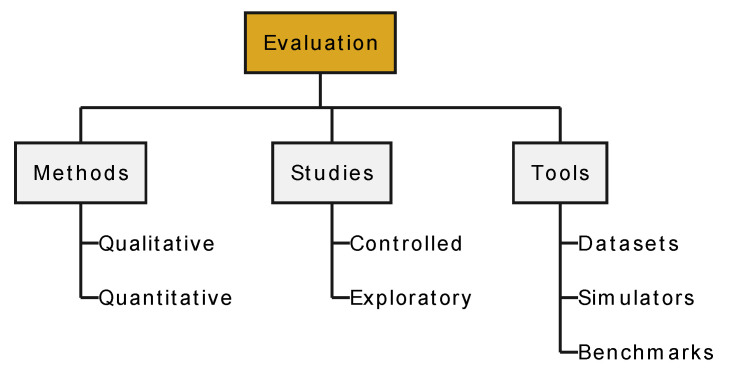
A taxonomy of evaluation for social robot navigation.

Evaluating social robot navigation systems is essential for gathering insights on comfort among users and optimizing their performance in real-world environments. This section discusses different evaluation methods, classifies types of studies conducted to explore or verify designed navigation algorithms, and identifies tools facilitating efficient assessment, namely datasets, simulators, and benchmarks Figure 9.

### 6.1. Methods

In general, evaluation methods encompass qualitative and quantitative approaches. Qualitative methods often involve subjective assessments, such as questionnaires conducted during user studies, which gauge users’ preferences and comfort levels while interacting with the robot (e.g., [9,40,87]). These subjective evaluations provide valuable insights into the social acceptability of robot navigation.

On the other hand, quantitative methods utilize objective metrics formulated mathematically to assess various aspects of robot performance and social awareness (e.g., [131,323,329,335,350]). These metrics enable precise assessment and, thus, evidence-based comparison of different navigation algorithms. Researchers employing a combination of qualitative and quantitative evaluation methods [85,131,328] can comprehensively gauge both the performance and suitability of human-aware navigation systems in meeting the expectations of users.

In recent work, Biswas et al. [33] stated that an ideal method of evaluating social robot navigation is a large-scale, costly, and time-consuming qualitative user study. However, due to the indicated drawbacks, automated methods that provide a quantitative approximation of facts are required. Quantitative assessment methods are particularly useful for learning-based approaches, where the reward of action must be numeric. Similarly, the authors of planners that employ heuristics or optimize a single criterion benefit from benchmarking their methods against various strategies. Since automated quantitative methods produce invariable indicators of the algorithm’s performance, they are particularly relevant for usage, e.g., during the new algorithm development stage. Nevertheless, grounding the social robot navigation requirements and approximating the social phenomena as quantitative metrics would be impossible without user studies yielding qualitative results.

### 6.2. Studies

Social robotics experiments often involve user studies to gather subjective human impressions about the robot’s behavior, which is crucial for social robot navigation as they provide valuable insights that can be directly transferred onto navigation system requirements Section 3. Experiments conducted for collecting such data can be differentiated between controlled and exploratory.

Controlled studies provide the possibility to conduct tests under configurable conditions. Hence, researchers can control variables and conditions to isolate specific factors, e.g., robot speed [60], passing distances [49], and observe their effects. This, in turn, allows for gathering more precise measures of robot behavior when operating with different navigation algorithms. This type of study might include both questionnaires and laboratory studies. In contrast, exploratory studies are conducted in natural conditions with minimum or no preparation. They might take the form of, e.g., a case study [354] to gain insights or field studies [1,2] connected with observing and gathering data (qualitative and/or quantitative) regarding a robot deployed in the target environment. The principles of human–robot interaction studies design were identified by Bartneck et al. in [355].

Controlled studies facilitate the systematic evaluation of the robot’s human awareness across different motion planning algorithms. However, direct comparison necessitates adherence to two crucial rules. Firstly, environmental conditions must be reproducible in subsequent trials. Secondly, a specific baseline motion planning setup (e.g., relying on classical navigation objectives), against which the examined navigation system will be compared, must remain unchanged in the following trials. In the literature, customized navigation approaches are contrasted against other algorithms [208] or a teleoperated agent [157], depending on the study design and goals.

Controlled laboratory studies intend to simplify complex interactions into prescribed scenarios of agents’ movements under constant environmental conditions, so the number of varying factors in subsequent trials is limited. Gao and Huang [5] identified standard scenarios investigated in social robot navigation works that include passing [60,320,356], crossing [71,206], overtaking [60,312,341], approaching [267,326,352], accompanying [119,157,245], or combined.

### 6.3. Tools

Multiple tools facilitate the evaluation of social robot navigation approaches. They are particularly useful for performing preliminary tests before arranging real-world experiments, which may pose a significant organizational effort [6,9,77,89].

#### 6.3.1. Datasets

The datasets can be employed to train models for human trajectory prediction and learning robot movements in populated environments. They are irreplaceable for neural approaches that optimize policy learning from data [269,322,348].

The pioneering datasets in the field are *ETH* [357] and *UCY* [358], suitable for tracking and prediction. They provide pedestrian trajectories from a top-view, fixed, outdoor-located camera. Later, Rudenko et al. [359] developed *THÖR* indoor dataset with human trajectory and eye gaze data with accurate ground truth information. The data were collected using motion capture hardware with 3D *LiDAR* recordings and a mobile robot in the scene. Another dataset, named *SCAND*, was proposed by Karnan et al. [337] and contains indoor and outdoor data from multiple sensors of a mobile robot teleoperated in a socially compliant manner.

Alternatively, *SocNav1* [360] and *SocNav2* [349] datasets were designed to learn and benchmark functions estimating social conventions in robot navigation by using human feedback in simulated environments. Wang et al. [361] developed *TBD* dataset containing human-verified labels, a combination of top-down and egocentric views, and naturalistic human behavior in the presence of a mobile capturing system moving in a socially acceptable way. Another dataset was used as a part of the *CrowdBot* project and is applicable for crowd detection and tracking, as well as learning navigation in populated, dynamic environments [362].

Recently, new datasets have emerged, for example, *SiT* [363], which contains indoor and outdoor recordings collected while the robot navigated in a crowded environment, capturing dense human–robot interactive dynamic scenarios with annotated pedestrian information. Nguyen et al. [364] developed *MuSoHu* dataset gathering recordings of sensors placed on human participants walking in human-occupied spaces; thus, interactions between robots and humans have not been captured. Hirose et al. [134] presented *HuRoN* dataset collected with multimodal sensory data from a robot operating with an autonomous policy interacting with humans in real-world scenes.

The publications relying on some of these datasets were identified in [5] and partially in [17], while in [3] the authors separated datasets for activity recognition, human pose estimation, and trajectory prediction.

#### 6.3.2. Simulators

In recent years, simulation experiments have been more often chosen due to the growth of the field of *RL* [147,154,174,341,345] and other data-driven approaches [184]. Simulators are particularly useful tools for the systematic evaluation of social robot navigation algorithms as they can provide identical initial conditions of experiments in the following trials, which is not always possible in user studies. Simulators also facilitate the agile development of algorithms and provide flexibility, which datasets often lack. Furthermore, as opposed to real-world tests, in terms of time and resources, they are easily reconfigurable and cost-effective in repeating trials.

Numerous simulation ecosystems have been developed for robotics [365]. The majority is directly applicable to social robotics as they provide movable human-like postures, and several are suitable for rich human–robot interaction. The main characteristics of state-of-the-art approaches for conducting virtual social robot navigation experiments are presented in Table 3, whereas Table 4 illustrates their methods for simulating human motion behaviors.

The comparison in Table 3 includes 2D and 3D simulators, as well as frameworks that have *ROS* integration (the most popular robotic framework), are actively maintained, and are open-source. Architectures of software for human simulation can be distinguished on standalone simulators and frameworks. The latter are usually designed for controlling simulated humans and they abstract from a specific simulator; therefore, interfacing components are necessary for integration. The proposed classification regards the fidelity of the replication of virtual robots, i.e., whether dynamic intricacies (friction, etc.) are included or only the ideal kinematic model is considered. Additionally, the comparison identifies the variety of tasks that can be performed by simulated humans and the methods for controlling humans. The capability of setting dynamic goals for virtual humans is crucial for rich human–robot interactions, which usually require an orchestrator. For example, handover tasks can be simulated only with the synchronization of human and robot activities. Specifically, the human receives an object after the robot approaches them (which in high-fidelity simulation always takes varying amounts of time); hence, the reception must be triggered at different timestamps.

On the other hand, Table 4 presents the characteristics of the virtual humans’ navigation in each simulation ecosystem. The comparison points out the algorithms used for motion planning and whether the motion of each agent can be configured differently. The classification also includes information on whether the simulation ecosystem allows the formation-like motion of virtual humans, which is restricted by the capabilities of motion planning algorithms available.

Notably, more advanced simulators facilitate transferring the algorithms from virtual to real-world hardware. All listed simulators except *flatland* (https://github.com/avidbots/flatland (accessed on 20 March 2024)) [345] provide the kinodynamic fidelity of robots, whereas the exactness of frameworks depends on the simulators they are integrated with. Simplified, lightweight simulators with the possibility to simulate dynamic agents, such as *SocialGym 2.0*, are well-suited for learning applications requiring multiple repetitions, whereas high-fidelity simulators, like *Gazebo* (*Ignition*) or *iGibson*, target the rich interaction scenarios. Nevertheless, transferring navigation methods from the simulation into real-world experiments is essential to demonstrate that developed algorithmic approaches work not only in simulated setups but are also reliable and prospective for wider applications.

#### 6.3.3. Benchmarks

Due to a growing set of navigation algorithms available, the importance of quantitative evaluation has increased. Lately, various automated quantitative assessment systems, called benchmarks, have been developed to ease the evaluation of traditional and social robot navigation. The appropriate benchmark design requires the knowledge of the requirements for robot navigation system Section 3, concurrently from the classical and human-aware points of view [76].

Several works have recently proposed benchmarking frameworks for evaluating robot motion planning algorithms from the classical navigation perspective [376,377,378,379,380,381,382,383,384,385], i.e., without considering human awareness constraints. These works mainly focus on performance metrics like navigation success rate, path length, or time required to reach the goal. Benchmarks for socially-aware robot navigation are the minority, but there are several works in that matter [33,369,386]. In some cases, simulators are coupled with internally calculated metrics for assessing navigation [369,374].

The primary features of state-of-the-art approaches for benchmarking robot navigation are presented in Table 5. The comparison includes only actively maintained and open-source benchmarks. The classification of methods focuses on the variety of metrics implemented (following the requirements taxonomy from Section 3), as well as determining suitable test environments (simulation/real world) and a set of analysis tools provided, e.g., for results presentation.

Quantitative metrics are the inherent parts of benchmark systems as they aim to implement objective criteria approximating subjective assessments. Therefore, the quantitative metrics should reflect mathematical formulas of requirements discussed in Section 3. Metrics covering most of the perceived safety principles for social robot navigation are developed in the *SRPB* (https://github.com/rayvburn/srpb (accessed on 20 March 2024)) benchmark, where human-awareness indicators also account for people tracking uncertainty, facilitating the evaluation with the robot’s onboard perception [76]. Besides the listed benchmark systems, several complementary indicators for assessing the perceived safety of humans in the context of social robot navigation also appear in [388]. The survey by Gao and Hoang [5] discusses in detail metrics presented in the literature.

## 7. Discussion

Although the literature regarding social robot navigation is vast, there are still issues of great significance that are fundamental for providing comprehensive social intelligence to robots. Major challenges and future work perspectives are identified in the remainder of this section.

### 7.1. In-Depth User Studies Exploring Human Preferences and Norm Protocols

The years 2000–2015 were very productive in user studies investigating social conventions and human preferences during interaction with robots [6,39,40,84,137]. Recently, we have observed much fewer exploratory and confirmatory studies [355], whereas, according to our extensive literature review, there are still some areas that could benefit from deeper investigation of how to obey complex norms and under what conditions Section 3.5. Also, multiple studies are contradictory regarding gaze modulation of robots Section 3.4.2. Continued research should provide valuable insights for understanding the robot’s social behavior requirements, as with the rapid growth of machine learning techniques, the analytical modeling of social phenomena receives less attention, being repressed by more accessible data-driven approaches.

### 7.2. Implementing Complex Social Conventions in Robot Navigation Systems

The classification of requirements’ fulfilment in various navigation approaches presented in Table 2 illustrates that social conventions are rarely addressed across algorithms and are rather implemented in a fragmentary manner. Among the specified in our taxonomy, the commonly neglected social norms include, e.g., standing in line or obeying elevator etiquette. We argue that the phenomenon of fewer works regarding social norm implementations is closely related to the necessity of including rich contextual information in robot navigation systems to behave in a socially acceptable way, which applies to the examples provided.

Multiple works discussed in Section 4.4 and Section 5 tackle contextual awareness fragmentarily, adhering only to specific rules to follow in a given context [131,220,221,222]. Notably, the literature review shows that many state-of-the-art *Deep Reinforcement Learning* methods implement rather a collision avoidance with dynamic objects than human-aware navigation, as the reward functions are formulated to consider only the separation distance between agents [134,146,174,342,343,344,345] imitating circular personal spaces, regardless of other social conventions and contextual cues.

A robot’s intelligence is often regarded as utilizing contextual information in its imperative [16,214]. Therefore, we argue that implementing complex social conventions in robot navigation systems requires integrating motion planning with knowledge bases [389], which could be updated by perception modules extracting environmental features in real time. However, including information from knowledge bases directly in existing motion planning approaches is impractical; hence, an additional component could be added to the standardized robot motion planning architecture consisting of a global path planner and a local trajectory planner. The role of the new *social activity planner* component would be to analyze environmental information and, based on the implemented social protocols, periodically generate new goal poses according to the task context Section 4.4.4. In this setup, the new component coordinates task execution in a socially acceptable manner, while the global path planner and the local trajectory planner handle motion planning concerning requirements related to the physical and perceived safety of humans, as well as to the robot’s motion naturalness. Additionally, the *social activity planner* component could be integrated with the robot’s head controller to properly modulate gaze direction during task execution.

An alternative method of integrating contextual richness directly into *DRL*-based end-to-end algorithms poses a possible challenge to capturing numerous intricacies of social robot navigation in a single control policy that might negatively affect the generalization capabilities of such approaches. Recently, a tendency to integrate learning-based approaches with classical algorithms evolved, e.g., [147,155,315,322], which might mitigate the identified drawback.

The concepts presented in [220,390] can be valuable insights for enhancing cognitive architectures that allow inferring environment objects’ relations once various facts about the environment, task, and humans are injected into the knowledge base. Works attempting to design context-aware social robot navigation integrated with a cognitive system are [228], where they used the *CORTEX* architecture [218], as well as [225,391]. Recently, the authors of [131] used socially aware navigation as one of the robot skills within a cognitive architecture, utilizing elements of environmental, interpersonal, and diversity contexts.

### 7.3. Context-Aware Framework for Modulating Motion Planning Objectives

Social robots are commonly deployed for tasks in complex environments. That requires rich contextual awareness, as the robots’ navigation objectives might vary according to a situation at hand Section 4.4.1. Enriched contextual awareness, discussed in Section 7.2, must be coordinated with a robot’s motion planning scheme to obtain human-aware behaviors and compliance with social conventions.

To achieve comprehensive human-aware robot navigation, which is a multiobjective problem, it is crucial not to treat social aspects as hard constraints. For instance, if a person is lying down due to fainting, the robot should be capable of approaching closely to check their condition, even if it means violating proxemics rules. Therefore, finding the relation between the navigation objectives and the contexts at hand could lead to obtaining more socially acceptable motions and enhance the perceived intelligence of a robot. This proposal aligns with one of the suggestions from [12].

Technically, the relation between contexts and navigation objectives can be reduced to a function that weighs the components of a multiobjective cost function designed to optimize human-aware navigation. Such a function could be embedded into the configurable context-aware orchestrating framework, which we indicate as a relevant future work perspective. Preliminary work in this matter has been conducted in [390], where the authors defined a mapping from the task-level knowledge to the motion-level knowledge to help enhance motion planning. Specifically, they identified variables that might be used in such an orchestrating framework and help dynamically weight the trajectory planning parameters. Nevertheless, finding the desired relation requires extensive user studies and creates perspectives for applying machine learning techniques, as manual tuning will probably be infeasible due to the complexity of the problem.

### 7.4. Context-Aware Benchmarks for Evaluating Nonprimitive Social Interactions

Benchmarks should also be aware of the contextual richness of the social robot navigation, as this would ease the assessment and deliver more accurate results. Contextual awareness of benchmarks is nontrivial to handle and infer from, while desired, similarly as in online navigation Section 7.3.

To exemplify the impact of environmental contexts, benchmark systems should only penalise the robot for traversing affordance spaces if they are actively exploited by humans, i.e., only if activity spaces were initiated. This, in turn, requires integrating multiple data during evaluation. The preliminary concept addressing the topic is implemented in *SEAN 2.0* simulator [369], which detects different social situations, but this information is not considered in metrics evaluation. In contrast, *SRPB* benchmark [76] regards the interpersonal context penalizing a robot for crossing through O-spaces of F-formations (human groups) while not considering environmental cues in metrics.

### 7.5. Design of Absolute Social Metrics for Social Robot Navigation Benchmarking

An essential need in quantitative benchmarking of social robot navigation is the design of absolute metrics, i.e., comparable between diverse scenarios. Most existing metrics do not sufficiently capture the generalizability of evaluated algorithms across diverse contexts [33,328,369,374,386]. This highlights the necessity of creating universal metrics that go beyond the specific context of individual scenarios. Standardized metrics applicable across various scenarios and study environments can enhance the reproducibility and transferability of findings.

## 8. Summary

In this paper, we grounded social robot navigation requirements based on the reviewed user studies regarding unfocused and focused human–robot interactions, which highlighted objectives on how robots should behave in populated environments. The human-aware robot navigation requirements are organized into our taxonomy consisting of requirements for ensuring the physical and perceived safety of humans, as well as the requirements assuring the robot’s motion naturalness and the robot’s compliance with the social norms. This classification is the basis for the analysis of algorithmic topics.

Our study examines the key methods for addressing the fundamental challenges of social robot perception, namely the detection and tracking of humans in the robot’s environment. Diverse environment representations utilized in different motion planning approaches were also discussed, as well as various methods for human trajectory prediction which is crucial in real robots equipped with sensors with a limited *field of view*. The survey also highlights the topic of contextual awareness and how it was tackled in state-of-the-art navigation approaches.

The major part of our review encompasses various methods employed for robot motion planning that take into account constraints arising from the presence of surrounding humans. Approaches present in the literature were classified into global path planning and local trajectory planning algorithms according to the common hierarchical structure of motion planning systems. Both global path planners and local trajectory planners were organized into groups sharing common algorithmic characteristics. Besides a thorough description of various navigation methods, these approaches are classified according to the established requirements taxonomy, based on the objectives addressed.

This survey also explores the methods for evaluating social robot navigation as well as study types and tools relevant to the agile development of navigation techniques. The tools for the assessment were discussed distinguishing datasets, simulators, and benchmarks. An extensive comparison of actively maintained simulators for social robotics was proposed. Moreover, benchmarks suitable for quantitative evaluation of social robot navigation were classified utilizing the proposed requirements taxonomy, according to the implemented metrics.

Our study examined state-of-the-art in the social robot navigation field and proposed several major topics for future work with a context-aware framework for modulating navigation objectives being the most promising. As a consequence of the rapidly growing field of social robot navigation, further integration of socially aware mobile robots in daily lives is expected. This cross-sectional review contributes to the broader understanding of social robot navigation fundamentals that lie on the border of robotics and social sciences. Our survey sheds light on social aspects that have not been adequately addressed in technical and social science papers.

## Figures and Tables

**Table 1:** A classification of literature reviews discussing social robot navigation. Typical taxonomy concepts were selected as grouping criteria. The classification identifies the main concepts investigated in each survey article according to the selected taxa.

Survey	Robot Types	Perception	Motion Planning	Evaluation	Nav. System Architecture
Kruse et al. [15]	wheeled	human traj. prediction	global cost functions, pose selection, global and local planning algorithms	simulation, user studies	allocation of main concepts
Rios-M. et al. [13]	—	social cues and signals	algorithms embedding social conventions	—	allocation of main concepts
Chik et al. [14]	wheeled	—	global path planning and local trajectory planning algorithms	—	various motion planning architectures
Charalampous et al. [16]	—	semantic mapping, human trajectory prediction, contextual awareness	—	benchmarks, datasets	—
Möller et al. [3]	—	active perception and learning, human behavior prediction	applications of activity recognition for path planning, trajectory modeling	benchmarks, datasets, simulation	—
Zhu and Zhang [18]	wheeled	—	DRL-based navigation algorithms	—	navigation frameworks structures
Mirsky et al. [4]	wheeled	—	navigation models and algorithms for conflict avoidance	simulation, various studies	—
Gao et al. [5]	—	—	models for assessment of specific social phenomena	questionnaires, various studies, scenarios, datasets, simulation, various metrics	—
Sánchez et al. [19]	—	human detection, semantic mapping, human motion prediction	predictive and reactive navigation methods	datasets	—
Mavrogiannis et al. [17]	design challenges	human intention prediction	extensive study involving various navigation algorithms	metrics, datasets, simulation, crowd models, demonstration, various studies	—
Guillén-Ruiz et al. [20]	—	classification of human motion prediction methods	agent motion models and learning-based methods, multi-behavior navigation	—	—
Francis et al. [12]	diversity of hardware platforms	predicting and accommodating human behavior	social navigation principles analysis, planning extensions with contextual awareness	methodologies and guidelines, metrics, datasets, scenarios, simulators, benchmarks	API for metrics benchmarking
Singamaneni et al. [11]	ground, aerial, aquatic	human intentions and trajectory prediction, contextual awareness	generation of global and local motion (planning, force, learning), identifying social norms	metrics, datasets, benchmarks, studies, simulators	—
Ours	ground, wheeled	human detection and tracking, trajectory prediction, contextual awareness	requirements-based global path and local trajectory planning methods with social constraints	metrics, datasets, benchmarks and simulators classification	—

**Table 2:** Classification of robot navigation methods implementing the requirements from the presented taxonomy.

Physical Safety	
[6,9,29,40,49,54,55,59,65,73,74,80,81,92,96,101,110,111,114,115,116,120,121,123,125,126,129,131,132,134,135,137,138,139,141,143,144,145,146,147,153,154,155,156,157,158,159,160,174,180,202,204,205,206,207,208,210,211,212,220,223,227,229,232,233,234,243,244,245,246,248,266,267,268,269,274,276,285,286,287,290,298,299,300,307,308,315,317,321,323,324,326,327,328,329,330,331,332,336,339,341,342,343,344,345,346,348,349,350,351,352]	
**Perceived Safety**	
Personal spaces	[9,29,49,54,59,65,73,74,80,81,101,120,123,125,129,131,132,134,137,141,143,144,145,146,147,156,157,158,159,160,174,205,206,207,210,212,220,223,232,233,234,244,245,246,266,267,268,269,286,287,290,299,300,307,315,317,326,327,329,342,343,344,345,346,348,349,352,353]
O-spaces of F-formations	[40,65,114,145,157,160,220,223,232,233,234,246,267,268,269,287,307,317,352,353]
Passing speed	[49,55,96,137,141,145,159,180,208,332]
Motion legibility	[55,74,101,139,141,147,159,160,180,202,206,207,208,317,321,328,336,346,350]
Approach direction	[6,40,54,80,81,92,157,229,244,245,246,267,269,286,307,326,332,352]
Approach speed	[40,54,81,92,157,245,246]
Occlusion zones	[132,141,266]
**Motion Naturalness**	
Velocity smoothness	[29,59,125,135,147,156]
Oscillations	[143,146]
In-place rotations	—
Backward movements	—
Gaze modulation	[73,96,101]
**Social Conventions**	
Accompanying	[40,110,111,114,115,116,120,121,126,132,157,174,229,243,244,245,246,308,329,330,331]
Affordance spaces	[123,125,223,227,267,268,307,352]
Activity spaces	[123,125,223,267,268,307,352]
Passing side	[49,59,73,132,137,221,336,341]
Yielding way	—
Standing in line	[125,129,220]
Elevator etiquette	—

**Table 3:** Classification of robotic simulation systems with capabilities for replicating human motion behavior. Abbreviations used in the table: *MG* stands for moving to a goal, *PG*—performing gestures, *FO*—following an object, *ST*—sitting, *CO*—conversating, *JG*—joining groups, and *MO*—moving to an object.

Approach	Software Architecture	Robot Fidelity	Human Task Variety	Human Control		
				**Scripted** **Scenarios**	**Dynamic** **Goals**	**Teleop**
*Webots* [366]	standalone	kinodynamic	*MG*	✓	—	—
*Gazebo* [367] (*Ignition*)	standalone	kinodynamic	*MG*, *PG*	✓	—	—
*PedsimROS* [140]	framework (*Gazebo* interface)	—	*MG*	✓	—	—
*flatland*	standalone	kinematic	*MG*	—	✓	—
*HuBeRo* [368]	framework (*Gazebo* interface)	—	*MG*, *PG*, *FO*, *ST*, *CO*, *MO*	✓	✓	✓
*SEAN 2.0* [369]	*Unity*	kinodynamic	*MG*, *JG*	✓	✓	✓
*Crowdbot* [370]	*Unity*	kinodynamic	*MG*	✓	—	—
*iGibson 2.0* [371]	standalone	kinodynamic	*MG*	✓	—	—
*InHUS* [372]	framework (*Stage*/*Morse* interfaces)	—	*MG*	✓	✓	✓
*IMHuS* [373]	framework (*Gazebo* interface)	—	*MG*	✓	✓	—
*SocialGym 2.0* [374]	framework (*UTMRS* interface)	kinodynamic	*MG*	✓	✓	—
*HuNavSim* [375]	framework (*Gazebo* interface)	—	*MG*	✓	✓	—

**Table 4:** Classification of robotic simulation systems from the perspective of methods to replicate human motion behavior.

Approach	Human Motion Planning	Human Motion Diversity	Human Groups
*Webots* [366]	naive trajectory following	configurable speed in a scripted trajectory	—
*Gazebo* [367] (*Ignition*)	*APF*-like	configurable weights of potentials	—
*PedsimROS* [140]	*SFM*	configurable motion model’s properties and group assignment	✓
*flatland*	any *ROS* plugin for motion planning	possible individual parameters for each planning agent	—
*HuBeRo* [368]	any *ROS* plugin for motion planning	possible individual parameters for each planning agent	—
*SEAN 2.0* [369]	*Unity*’s built-in path planner with *SFM*	configurable behaviors (randomized, handcrafted or graph-based control of pedestrians), variable posture	✓
*Crowdbot* [370]	*DWA*, *RVO*, *SFM*	configurable speed in a scripted trajectory	—
*iGibson 2.0* [371]	$A^{*}$ with ORCA	configurable object radius of ORCA	—
*InHUS* [372]	any *ROS* plugin for motion planning	possible individual parameters for each planning agent	—
*IMHuS* [373]	any *ROS* plugin for motion planning	possible individual parameters for each planning agent	—
*SocialGym 2.0* [374]	*SFM*	configurable motion model’s properties and group assignment	—
*HuNavSim* [375]	*APF*-like/*SFM*	configurable behaviors (regular, impassive, surprised, curious, scared, threatening)	✓

**Table 5:** A classification of state-of-the-art methods for quantitative evaluation of robot navigation requirements. The number of ticks (✓) reflects the number of metrics implemented in each benchmark. Abbreviations used: *S* stands for simulation environments, *R*—real-world environments, and *S/R* reflects simulation and real-world environments.

Name	Metrics					Suitable Env.	Analysis Tools
	**Classical** **Navigation** **Performance**	**Physical** **Safety**	**Perceived** **Safety**	**Motion** **Naturalness**	**Social** **Norms**		
*iGibson* *Benchmark* [387]	✓	—	✓	—	—	*S*	–
*MRPB* [382]	✓✓✓✓	✓	—	✓	—	*S/R*	–
*BenchMR* [376]	✓✓✓✓✓ ✓	✓	—	✓	—	*S*	scenario rendering, metrics plots
*CrowdBot* *Benchmark* [370]	✓✓	✓✓	—	✓✓✓✓	—	*S*	scenario rendering, metrics plots
*SocNavBench* [33]	✓✓✓✓✓ ✓✓✓✓✓	✓✓	✓✓	✓✓	—	*S*	scenario rendering, metrics plots
*Arena-Bench* [383]	✓✓✓✓✓ ✓✓✓	✓	—	✓✓✓	—	*S*	scenario rendering, metrics plots
*SEAN 2.0* [369]	✓✓✓✓✓ ✓✓✓	✓	✓✓	✓	—	*S*	–
*InHuS* [372]	✓	✓✓	✓	—	—	*S/R*	scenario and metrics rendering
Tafnakaji et al. [385]	✓✓✓✓✓	—	—	✓	—	*S/R*	scenario rendering
*SRPB* [76]	✓✓✓✓✓ ✓	✓✓✓✓	✓✓✓✓✓ ✓✓✓✓✓ ✓✓✓✓✓ ✓	✓✓✓✓✓	—	*S/R*	scenario rendering, metrics plots, exporting results to a L^A^T_E_X table or a spreadsheet
*HuNavSim* [375]	✓✓✓✓✓ ✓✓✓	✓✓✓✓	✓✓✓✓	✓✓	—	*S*	—

## Data Availability

The data presented in this study are available on request from the corresponding author.
